# Genomic diversity and epidemiological significance of non-typhoidal *Salmonella* found in retail food collected in Norfolk, UK

**DOI:** 10.1099/mgen.0.001075

**Published:** 2023-07-31

**Authors:** Samuel J. Bloomfield, Nicol Janecko, Raphaёlle Palau, Nabil-Fareed Alikhan, Alison E. Mather

**Affiliations:** ^1^​ Quadram Institute Bioscience, Norwich Research Park, Norwich, UK; ^2^​ University of East Anglia, Norwich, UK

**Keywords:** *Salmonella*, genomics, antimicrobial resistance, food

## Abstract

Non-typhoidal *Salmonella* (NTS) is a major cause of bacterial gastroenteritis. Although many countries have implemented whole genome sequencing (WGS) of NTS, there is limited knowledge on NTS diversity on food and its contribution to human disease. In this study, the aim was to characterise the NTS genomes from retail foods in a particular region of the UK and assess the contribution to human NTS infections. Raw food samples were collected at retail in a repeated cross-sectional design in Norfolk, UK, including chicken (*n*=311), leafy green (*n*=311), pork (*n*=311), prawn (*n*=279) and salmon (*n*=157) samples. Up to eight presumptive NTS isolates per positive sample underwent WGS and were compared to publicly available NTS genomes from UK human cases. NTS was isolated from chicken (9.6 %), prawn (2.9 %) and pork (1.3 %) samples and included 14 serovars, of which *Salmonella* Infantis and *Salmonella* Enteritidis were the most common. The *S*. Enteritidis isolates were only isolated from imported chicken. No antimicrobial resistance determinants were found in prawn isolates, whilst 5.1 % of chicken and 0.64 % of pork samples contained multi-drug resistant NTS. The maximum number of pairwise core non-recombinant single nucleotide polymorphisms (SNPs) amongst isolates from the same sample was used to measure diversity and most samples had a median of two SNPs (range: 0–251). NTS isolates that were within five SNPs to clinical UK isolates belonged to specific serovars: *S*. Enteritidis and *S*. Infantis (chicken), and *S*. I 4,[5],12:i- (pork and chicken). Most NTS isolates that were closely related to human-derived isolates were obtained from imported chicken, but further epidemiological data are required to assess definitively the probable source of the human cases. Continued WGS surveillance of *Salmonella* on retail food involving multiple isolates from each sample is necessary to capture the diversity of *Salmonella* and determine the relative importance of different sources of human disease.

## Data Summary

The sequence data generated in this study are available in the Sequence Read Archive (SRA; https://www.ncbi.nlm.nih.gov/sra) under project PRJNA939716.

**Impact Statement**

Non-typhoidal *Salmonella* (NTS) is a major cause of diarrhoea and can be found on food. This study describes NTS isolates collected from retail food from the UK using whole genome sequencing (WGS) and compares the isolates to those collected from humans in the UK to determine the degree to which these foods may contribute to human NTS infections. We isolated NTS from chicken, pork and prawns, but only the isolates from chicken and pork contained antimicrobial resistance determinants. NTS isolates collected from the same food sample varied in diversity, which has implications for outbreak analyses which typically analyse one NTS isolate per food sample. Fourteen serovars of NTS were isolated, but only three consisted of isolates that were closely related to human-derived UK isolates: *Salmonella* Enteritidis and *Salmonella* Infantis (chicken), and *Salmonella* I 4,[5],12:i- (pork and chicken). Most NTS isolates that were closely related to human-derived isolates were obtained from imported chicken, but further epidemiological data are required to assess definitively the probable source of the human cases. Continued WGS surveillance of *Salmonella* on retail food involving multiple isolates from each food sample is necessary to determine the diversity of *Salmonella* present and the relative importance of different sources of human disease.

## Introduction

Non-typhoidal *Salmonella* (NTS) includes all serovars of *Salmonella enterica* subspecies *enterica* not associated with typhoid or paratyphoid fever. The majority of illnesses caused by NTS manifest as gastroenteritis, but it can also cause systemic infections. Salmonellosis is a worldwide problem [1], and in the European Union (EU) it is the second most common cause of bacterial gastroenteritis [2]. In the UK, the annual cost of foodborne disease from *Salmonella* is estimated to be £212 million, with more than 31 000 food-related cases and 2000 hospitalizations per year [3].

NTS infections are usually self-limiting but antimicrobial treatment may be required for immunocompromised patients or invasive infections [4]. The proportion of NTS that are resistant or multi-drug resistant, that is resistant to three or more different classes of antimicrobials, has increased worldwide over the last three decades [5–7]. Antimicrobial resistance (AMR) can also spread between different bacterial strains and species through horizontal gene transfer [8]. Therefore, preventing the spread of AMR in NTS is important for the treatment of invasive salmonellosis and potentially future infections caused by other bacteria.

NTS can be typed based on their lipopolysaccharide (O), flagellar (H) and capsule (Vi) antigens into serovars, most of which form discrete phylogenetic clades separate from other serovars [9]. Animals and animal products are the main sources of known NTS outbreaks [10], and some serovars are associated with specific animal sources, such as *Salmonella* Enteritidis and poultry products; for other serovars, the association is restricted to certain countries, such as a link between *S*. Sofia and poultry in Australia [11]. Typical diets vary between countries around the world and are continually changing [12], as are food supply trading partners [13], altering the types and sources of foods with which humans interact. In addition, there is evidence of *Salmonella* transmission between humans [14]. Highly discriminatory tests are required to distinguish between the strains from clinical cases and potential sources to determine likely sources of infection, and therefore identify where interventions could be applied.

Whole genome sequencing (WGS) involves determining the entire genome of an organism. Genomic analysis has allowed the distinction between bacterial isolates that were previously indistinguishable using phenotypic and other molecular methods [15]. As the costs of this approach have decreased, countries have begun routinely using WGS on NTS isolates collected from clinical cases and other sources to identify clusters of isolates that are closely related and may represent outbreaks [16]. In England and Wales, 95 % of NTS isolates from human clinical cases identified by diagnostic laboratories are sent to the United Kingdom Heath Security Agency (UKHSA), as are some from food and environmental sources, which are whole genome sequenced and made publicly available [17]. This has helped determine when specific NTS strains were introduced into the UK [18] and the origin of specific outbreaks [19]. However, surveillance programmes typically obtain a single isolate from a given sample. Sources can contain diverse strains of NTS but few studies have investigated the diversity of NTS isolates from different commodities and those that do predominantly only investigate down to the serovar level [20]. Recently, an investigation into an *S*. Enteritidis outbreak in the UK associated with ready-to-cook chicken and turkey products revealed seven samples where two different serovars were identified and sequenced [19], with the most common serovar detected being *S*. Infantis. The outbreak-related *S*. Enteritidis strains were of lower abundance within the samples and were only detected following a serovar-specific approach. There are implications for outbreak analyses as only testing a single NTS isolate may not capture the full NTS population diversity on the sample and hinder investigations into potential sources of infection.

In this study we investigated the potential risk of NTS exposure from different food commodities in the UK by sequencing the genomes of NTS isolates collected from retail food in Norfolk [21], investigating the number of AMR determinants present, the genetic relatedness to clinical isolates and the diversity of isolates on the food specimens.

## Methods

### Sampling

A population- and market-share-weighted longitudinal repeated cross-sectional study was used to collect raw chicken (*n*=311), leafy green (*n*=311), pork (*n*=311), prawn (*n*=279) and salmon (*n*=157) products at retail in Norfolk, UK, between 21 May 2018 and 25 November 2019, as previously described [21] (Table S1, available in the online version of this article). A cold chain was maintained whilst transporting food from retail stores to the testing laboratory at Quadram Institute Bioscience, Norwich, UK. The meat and seafood samples consisted of a variety of cuts and the leafy green samples consisted of a range of combinations of plants. Each sample underwent enrichment and culture for NTS as described by Janecko, Zamudio *et al*. [21].

Briefly, for each sample, 100 g of sample was placed into a filter blender bag (Corning) with 225 ml of buffered peptone water (BPW) (Southern Group Laboratory (SGL)) and was stomached for 30 s at 100 rpm. The stomached sample was incubated for 18±3 h at 37 °C. One millilitre of incubated BPW was added to 10 ml of Muller-Kauffman tetrathionate-novobiocin (MKTTn) broth (Oxoid) and incubated at 37 °C for 24±3 h, and 0.1 ml of incubated BPW was inoculated onto modified semi-solid Rappaport Vassiliadis (MSRV) agar (Oxoid) as three equally spaced spots and incubated at 42 °C for 24±3 h. For MKTTn media, a 10 µl loop of the incubated media was inoculated onto a split xylose-lysine-deoxycholate/brilliance *Salmonella* agar (XLD/BSA) plate (Oxoid) and incubated at 37 °C for 24±3 h. For MSRV agar, if *Salmonella *-like migration (cream-coloured, smooth) was observed then a 10 µl loop of the outer portion of migratory growth was inoculated onto XLD/BSA plates and incubated at 37 °C for 24±3 h. If no *Salmonella *-like migration was observed, then the MSRV plates were incubated at 42 °C for an additional 24±3 h and observed for *Salmonella *-like migration. XLD/BSA plates were observed for *Salmonella *-like colonies (colonies with black centres on XLD and purple colonies on BSA) and up to two from each agar plate were subcultured onto MacConkey agar (MAC; Oxoid) to capture NTS diversity in case they were only isolated using one method, streaked for isolated colonies and incubated at 37 °C for 24±3 h. *Salmonella *-like colonies on MAC (non-lactose fermenting) were subcultured onto tryptic soy agar (TSA; SGL), streaked for individual colonies and incubated at 37 °C for 24±3 h. TSA colonies were subcultured onto urease slopes (Oxoid) and incubated at 37 °C for 24±3 h, were tested using indole spot reagent (Remel), and were tested for agglutination with *Salmonella* O antiserum poly A-I and Vi (BD). Isolates that were indole- and urease-negative, and that agglutinated with antisera were presumptively classified as representing *Salmonella* and underwent WGS.

### Whole genome sequencing

Genomes were extracted using the Maxwell RSC Cultured Cells DNA Kit (Promega). Libraries were created using the Nextera XT DNA Library Preparation Kit (Illumina) and sequenced on a NextSeq 550 System (Illumina) as 150 bp paired-end reads.

### Whole genome analysis

Genomic analyses were performed on the Cloud Infrastructure for Microbial Bioinformatics (CLIMB) server [22]. Illumina reads were trimmed using Trimmomatic v0.36 [23] (Supplementary material). Trimmed reads were assembled using Spades v3.11.1 [24] in ‘careful’ mode. The quality of the assemblies was assessed using QUAST v4.6.3 [25] and CheckM v1.1.2 [26] and by aligning reads to the assemblies using the Burrows–Wheeler aligner (BWA) v0.7.17 [27]. Assemblies were accepted if they consisted of fewer than 500 contigs that were over 500 bp, fewer than 50 duplicate genes and had a mean read depth of the four largest contigs above 30. SISTR v1.1.1 [28] was used to identify the serovars of the *Salmonella* genomes.

Prokka v1.13 [29] was used to annotate assemblies. Roary v3.13.0 [30] was used to cluster annotated assemblies with a 95 % amino acid identity and core gene threshold and form a core gene alignment. RaxML v8.2.4 [31] was used to generate a maximum-likelihood tree based on single nucleotide polymorphisms (SNPs) in this core gene alignment. The process was repeated with *Escherichia coli* (SAMN02604091) to root the tree.

For each serovar identified, Snippy v3.2 (https://github.com/tseemann/snippy) was used to align reads to representative reference genomes (Table S2). Gubbins v2.3.1 [32] was used to remove SNPs putatively associated with recombination before the number of SNPs between isolates from the same sample was quantified.

To determine if the enrichment methods, culture methods or combinations of these methods were more effective at detecting the diversity of NTS on food samples, the maximum number of SNPs between NTS isolates collected from the same sample belonging to the same serovar using the same methods was calculated. Kruskal–Wallis tests were used to compare the culture and enrichment methods. Statistical analyses were performed using R v3.6.1 [33].

AMR genes and plasmid replicons were identified using ARIBA v2.14.4 [34] and the NCBI AMR [35] and PlasmidFinder [36] databases, respectively. AMRFinder v0.4.0 [35] was used to identify point mutations associated with AMR. Isolates were classified as multi-drug resistant (MDR) if they contained AMR determinants that encoded resistance to three or more antimicrobial agent classes. ARIBA was also used to determine the sequence type (ST) of genomes using the *Salmonella* database on PubMLST [37].

### *S.* Infantis genome analysis

For isolates identified as *S.* Infantis, the pESI plasmid replicon was searched for using Abricate v0.7 (https://github.com/tseemann/abricate) and the PlasmidFinder database for assembled contigs. BWA was also used to align trimmed reads to the pESI plasmid sequence CP070303, and samtools v1.8 [38] was used to determine the proportion of the sequence with a read depth of 10 or higher (i.e. coverage). Metal-tolerance genes were identified in *S.* Infantis genomes using tBLASTn v2.7.1 [39] and the BacMet v2.0 [40] database with 95 % identity and coverage cut-offs.

### Comparative analysis with publicly available UK genomes

The EnteroBase [41] *Salmonella* database was used to search for all genomes that were collected from the UK between 1916 and 2021 to determine how the isolates collected from food related to those isolated from humans in the UK, where the food products were consumed. For each serovar that was identified in at least one food sample in this study, a representative of each hierarchical clustering 50 (HC50) group was downloaded, assembled using Spades, and the distance between each representative and those genomes collected from food was calculated using Mash v2.0 [42] using default settings. For the 14 serovars investigated in this study, each contained between three and 314 HC50 groups. For the HC50 representative that was most closely related to the food isolate(s), all UK genomes belonging to that HC50 were downloaded and analysed further. If fewer than ten genomes belonged to the HC50, then a higher hierarchical cluster group was analysed (e.g. HC100). However, if the group consisted of more than 1000 genomes then the process was repeated on a lower hierarchical cluster group (e.g. HC10). For all genomes downloaded, paired reads were processed and assembled as previously stated to determine their quality. For each serovar, the SNP and maximum-likelihood tree analyses were repeated with the isolates collected from this study and those from EnteroBase. For human-derived UK isolates that contained the fewest number of core non-recombinant SNPs compared to isolates collected from this study, AMR determinants and plasmid replicons were identified using ARIBA as stated above.

### Between food commodity sample comparison

For the NTS isolated in this study from food, the serovars to which they belonged, the number of AMR determinants identified and the distance to the closest human isolate were compared amongst food samples collected from different commodities. For chicken samples, food origin, food storage and type of shop from which it was purchased were also compared. Fewer than ten samples from each of the other commodities tested positive for NTS so further metadata comparisons were not investigated on them. The season in which the food samples were obtained was previously not found to influence the incidence of NTS, so this was not investigated further [21].

## Results

### Samples

NTS was isolated from 42 food samples and 199 isolates from these samples were sequenced using short-read sequencing. Of the five food commodities analysed, 9.6 % (30/311) of raw chicken and 1.3 % (4/311) of raw pork samples tested positive for NTS. The prawn samples consisted of 217 raw samples, 3.7 % (8/217) of which tested positive for NTS, and 62 cooked samples, none of which tested positive for NTS. No raw salmon or leafy green samples tested positive for NTS [21].

Chicken samples were classified based on packaging information, as follows: ‘domestic’ if source was the UK (*n=*214), ‘imported’ if source was from outside the UK (*n=*88), ‘mixed’ if source consisted of the UK and another country (*n=*1), and ‘unknown’ if the origin of the food was not clear (*n=*8). Previous analysis found that imported chicken was associated with NTS [21].

### Serovars

**Fig. 1.**
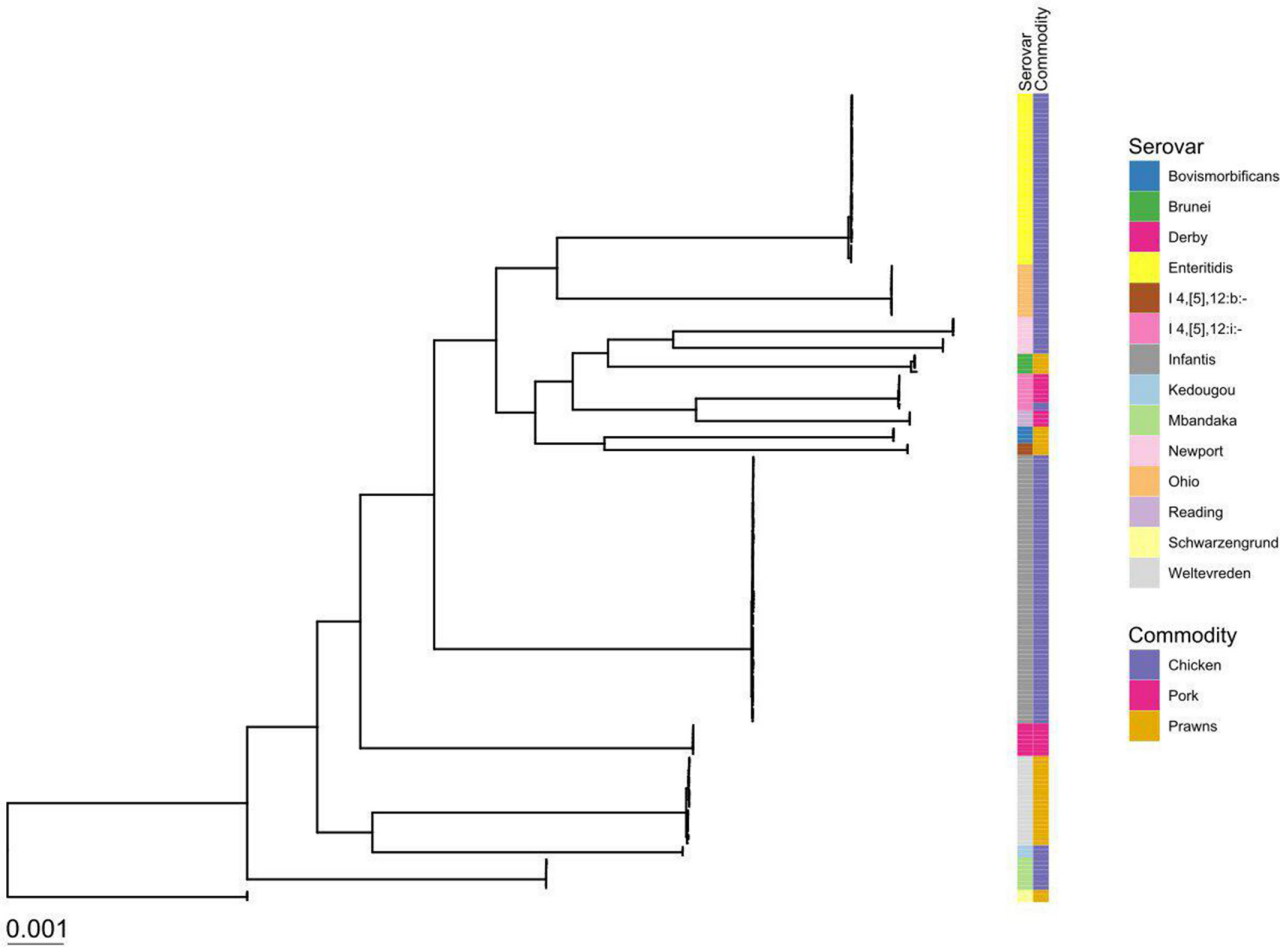
Maximum-likelihood tree of 199 food-derived NTS genomes coloured by serovar and the food commodity from which they were isolated (source). The phylogenetic branch lengths are given in nucleotide substitutions per site, and therefore a branch of length 0.001 (as represented by the bar) equates to 3352 substitutions, given that the core gene alignment consisted of 3 352 337 bp.

Fourteen *Salmonella* serovars were isolated from the food samples. The serovars were largely food-commodity-specific, apart from *S.* I 4,[5],12:i:- that was found in both pork and chicken samples (Fig. 1).

For chicken samples, *S*. Newport (*n*=2 samples) and *S.* Enteritidis (*n*=9 samples) were only isolated from imported samples, whilst *S.* I 1,4,[5],12:i:- (*n*=1 sample), *S*. Kedougou (*n*=1 sample), *S*. Mbandaka (*n*=1 sample) and *S*. Ohio (*n*=2 samples) serovars were only isolated from domestic samples (Fig. S1). The *S*. Infantis (*n*=14 samples) serovar was isolated from both domestic and imported chicken samples. Sample storage and sample origin were strongly linked in chicken in this study, with 85 % of imported chicken samples available as frozen compared to 1 % of domestic samples available as frozen. The exception was one chilled imported sample from which *S.* Enteritidis was isolated. The only serovar isolated from chicken samples obtained from butcher shops was *S*. Infantis.

### Antimicrobial resistance

**Fig. 2.**
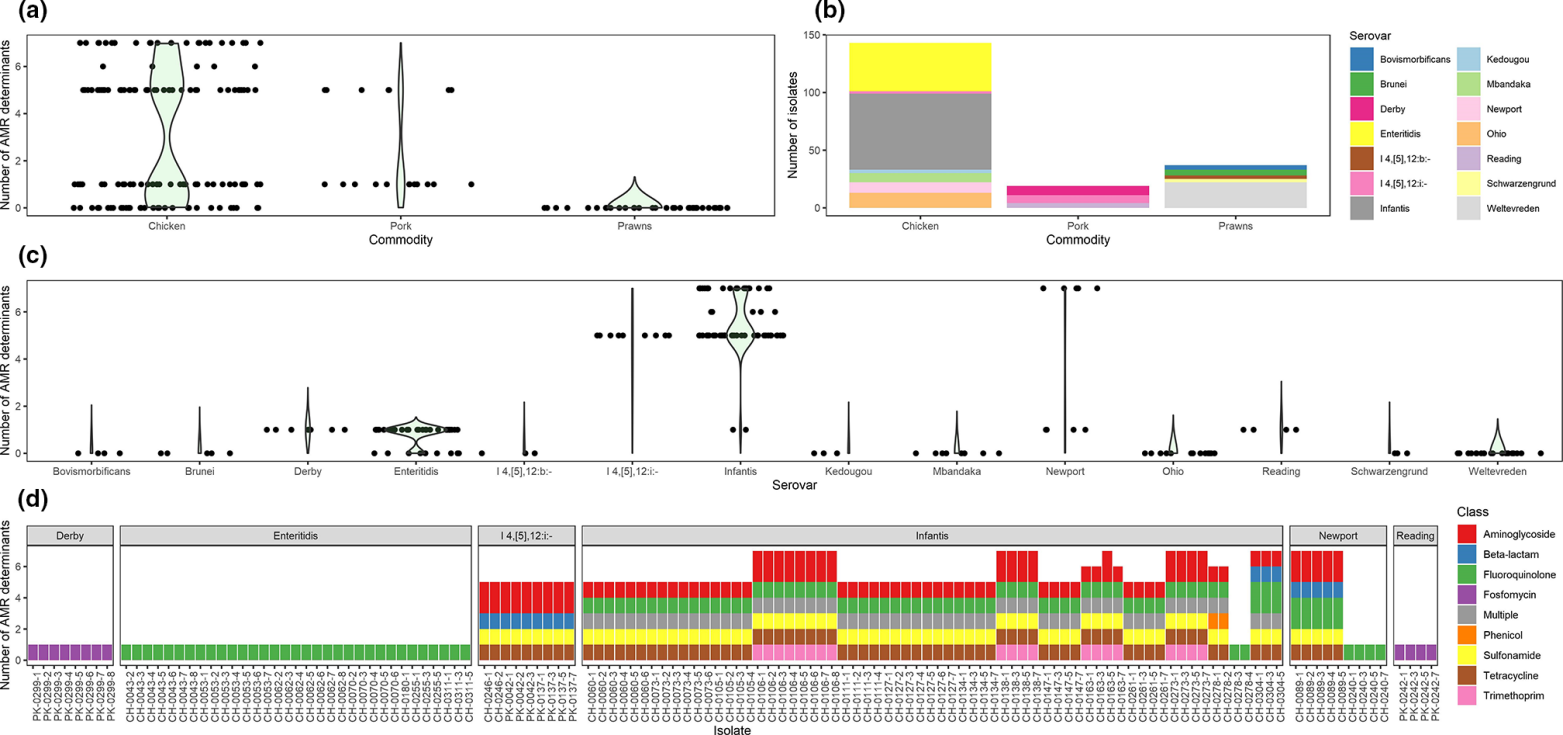
Jitter and violin plot of the number of AMR determinants identified in the NTS from different food commodities (**a**); bar graph of the number of isolates collected from each food commodity (source) coloured by serovar (**b**); jitter and violin plot of the number of AMR determinants identified in the NTS from different serovars (**c**); and bar graphs of the number of AMR determinants found in NTS isolates containing one or more AMR determinants and coloured by the antimicrobial agent to which they encode resistance (**d**).

AMR analysis found that 129 of the 199 (64.8 %) *Salmonella* isolates contained at least one AMR determinant, 70 (35.2 %) contained no AMR determinants and 78 (39.2 %) were classified as MDR (Fig. 2a, Table S3). Of the 14 serovars to which these isolates belonged, AMR determinants were found in six and MDR isolates were found in three (Fig. 2b, c). MDR NTS isolates belonged to the *S.* I 4,[5],12:i:-, *S.* Infantis and *S.* Newport serovars (Fig. 2d).

Of the 42 samples that tested positive for NTS, 29 contained at least one AMR NTS isolate and 18 contained at least one MDR NTS isolate. No samples contained a mixture of AMR and non-AMR NTS isolates, but a single chicken sample contained a mixture of both MDR and non-MDR AMR isolates.

For NTS-containing chicken samples, 80 % (24/30) contained at least one AMR determinant-containing NTS isolate and 53 % (16/30) contained at least one MDR NTS isolate (Fig. S2). For the four NTS-containing pork samples, all contained at least one AMR determinant-containing NTS isolate and half (2/4) contained at least one MDR NTS isolate. None of the NTS isolates from prawns contained any AMR determinants.

### *S.* Infantis genomes

*S.* Infantis was the most commonly isolated serovar in this study and was only isolated from chicken (Fig. 1). All *S.* Infantis isolates were ST 32, which is associated with the pESI plasmid that contains an IncFIB plasmid replicon [43]. Abricate identified the IncFIB(K)_1_Kpn3 plasmid replicon in all these genomes with a 99.6 % identity and 82.8 % coverage. ARIBA was unable to detect this plasmid replicon with a 90 % identity cut-off. The read coverage of *S.* Infantis genomes to the pESI reference sequence, CP070303, was 73.4–86.0 % (Fig. S3). The AMR and metal-tolerance genes of the pESI plasmid are usually located in two regions [18]. Regarding region 1, 26 % (17/66) of *S.* Infantis isolates in this study contained the *aph(3)-Ia* gene and 3 % (2/66) contained the *floR* gene associated with this region, but none contained any of the other genes associated with region 1: *aac(3)-IV*, *aph(4)-Ia*, *ars*, *blaCTX-M-65*, *dfrA-14* and *fosA3*. Regarding region 2, 97 % (64/66) of *S.* Infantis isolates in this study contained the genes associated with this region: *aadA1*, *mer*, *sul1* and *tetA*.

### Diversity

Of the 42 food samples from which NTS was cultured, 40 contained isolates belonging to a single serovar; one prawn sample contained isolates that belonged to two serovars (PR-0007) and one prawn sample contained isolates that belonged to three serovars (PR-0151). For PR-0007, *S*. Weltevreden was isolated using the MKTTn-BSA (*n*=2 isolates) and MKTTn-XLD (*n*=2 isolates) methods, and *S*. Bovismorbificans was isolated using the MSRV-BSA (*n*=2 isolates) and MSRV-XLD (*n*=2 isolates) methods. For PR-0151, *S*. Brunei was identified using the MKTTn-BSA (*n*=1 isolate) method, *S.* Weltevreden was isolated using the MSRV-BSA (*n*=1 isolate) and MSRV-XLD (*n*=1 isolate) methods, and *S*. I 4,[5],12:b:- was isolated using the MKTTn-BSA (*n*=1 isolate), MSRV-BSA (*n*=1 isolate) and MSRV-XLD (*n*=1 isolate) methods.

Comparisons between the isolates belonging to each serovar and the methods used to detect them found that all 14 serovars were detected by the BSA and XLD culture methods, and MSRV enrichment, but the *S*. Bovismorbificans serovar was not isolated using mKTTN enrichment. Comparisons between isolates belonging to each sample and method of detection found that the BSA culture method detected NTS from 42 positive samples, but only 40 cultured for NTS using the XLD culture method, 39 by the mKTTN enrichment method and 37 by the MSRV method.

**Fig. 3.**
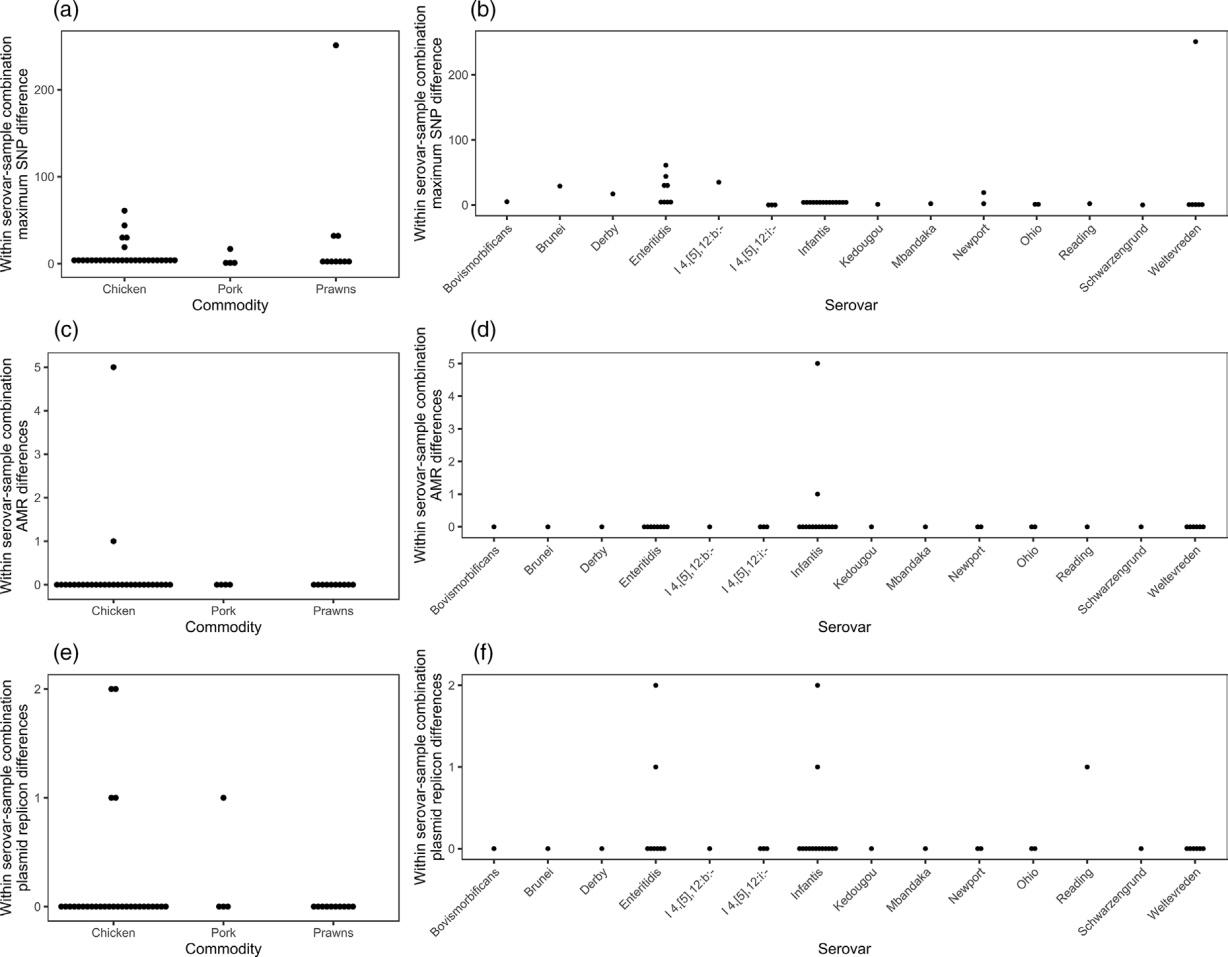
Dot plots of the maximum core non-recombinant SNP difference (**a and b**), AMR determinant differences (**c and d**) and plasmid replicon differences (**e and f**) between isolates belonging to the same serovar from the same sample and separated by food commodity (**a, c, e**) and serovar (**b, d, f**).

NTS diversity amongst isolates collected from the same sample and serovar (hereafter called a serovar–sample combination) was measured by calculating the maximum number of pairwise core non-recombinant SNPs amongst these isolates. Most isolates from the same serovar–sample combination were similar with a median of two pairwise SNP differences (interquartile range: 0–6 SNPs). In total, 26 % (11/42) of NTS-positive samples contained isolates that differed by five or more SNPs, with one prawn sample containing *S*. Weltevreden isolates that differed by up to 251 SNPs (Fig. 3). There were also differences in the accessory genomes of isolates of the same serovar–sample combination: *S.* Infantis isolates from two chicken samples differed in the presence of AMR determinants and *S.* Enteritidis isolates from two chicken samples, *S.* Infantis isolates from two chicken samples and *S.* Reading isolates from a pork sample differed in the presence of plasmid replicons.

To determine if any of the methods were better at detecting NTS diversity on food samples, the maximum number of pairwise core non-recombinant SNPs between isolates collected from the same serovar–sample combination using the same method were calculated (Fig. S4). MSRV identified a larger number of SNPs than MKTTn (*P*=8.7×10^−3^), but the culture method (*P*=0.87) was not associated with larger numbers of SNPs.

### Comparison with human-derived isolates

**Fig. 4.**
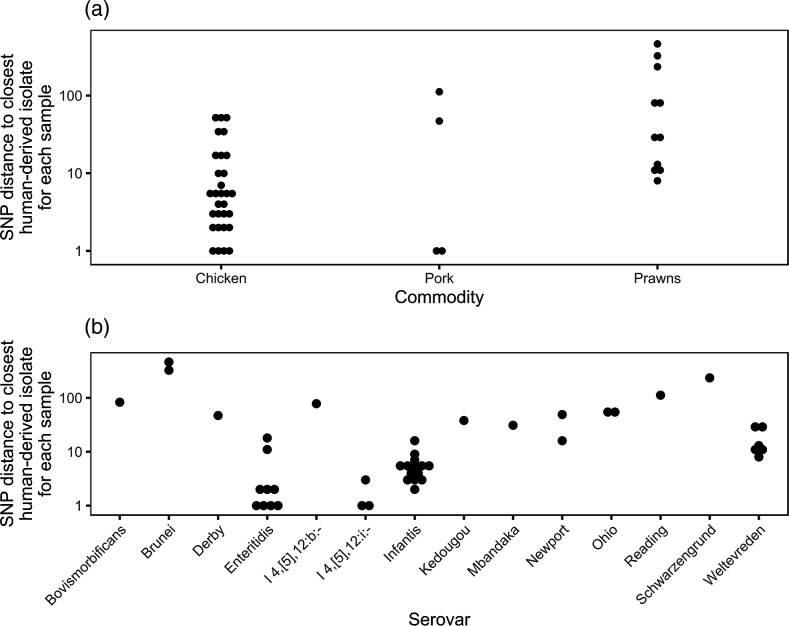
Dot plots of the SNP distance to the closest publicly available UK human-derived isolate for each NTS-positive sample, separated by food commodity (**a**) and serovar (**b**).

The isolates collected from food in this study were 0–462 (median=5.5) core non-recombinant SNPs from the closest UK human-derived isolate available (Figs 4 and S5–S18). As the UKHSA uses five SNPs for identifying outbreaks of salmonellosis [17], this cut-off was used to identify food isolates that could represent strains associated with human clinical infections. Of the 42 food samples that tested positive for NTS, 21 contained isolates that shared fewer than five SNPs with the closest UK human-derived isolate, and these comprised 63 % (19/30) of NTS-positive chicken, 50 % (2/4) of NTS-positive pork and 0 % (0/8) of NTS-positive prawn samples. Taking NTS culture positive and negative samples into consideration, 6.1 % (19/311) of chicken, 0.6 % (2/311) of pork and 0 % (0/217) of raw prawn samples contained isolates that shared fewer than five SNPs with the closest UK human-derived isolate.

For the 21 food samples with closely related isolates to those collected from UK human sources, the closest human isolates were collected between three years before or one year after the food samples, and for 13 samples the food and closest human isolate were collected during the same year (Fig. S19). For two of these 21 samples the closest UK human isolate differed by a plasmid replicon, and for one of these samples the closest UK human isolate differed by an AMR determinant. However, the closest human UK isolate for the remaining 18 samples contained identical plasmid replicons and AMR determinants to the closest food isolate.

Of the 88 imported chicken samples investigated, 17 % (95 % confidence interval (CI): 9.2–25 %) contained NTS isolates that were within five SNPs to clinical isolates compared to 2.3 % (95 % CI: 0.31–4.4 %) of the 214 domestic samples investigated, and these percentages were significantly different (*P*<0.00001) (Fig. S20). Of the 225 chilled chicken samples, 2.7 % (95 % CI: 0.56–4.8 %) contained NTS isolates that were within five SNPs to clinical isolates compared to 15 % (95 % CI: 7.5–23 %) of the 86 frozen chicken samples investigated, and these percentages were significantly different (*P*=5.3×10^−5^).

The pairwise SNP distances between food samples and the closest UK human isolate were serovar dependent, with 78 % (7/9) of *S.* Enteritidis, 100 % (3/3) of *S.* I 4,[5],12:i- and 93 % (11/14) of *S.* Infantis samples sharing fewer than five SNPs with the closest human isolate. If the SNP cut-off was increased from five to ten SNPs between food- and UK human-derived isolates, some *S.* Weltevreden from prawn samples would also have been classified as being closely related to a UK human-derived isolate.

## Discussion

NTS infections cause the second highest burden of bacterial gastroenteritis in the UK, contributing to significant health and economic costs [3]. As most cases of *Salmonella* are sporadic in nature and cannot be attributed to any specific source [44], it is important to investigate potential sources of NTS with the highest resolution techniques available [45, 46].

WGS is the most discriminatory method available to compare NTS isolates and has been used to identify and analyse outbreaks that were undetectable using previous methods [16]. Many countries have incorporated routine WGS of NTS into their surveillance programmes, which have been used to identify outbreaks [17, 47]. In this study, we sequenced up to eight isolates per sample to understand the genetic diversity of *Salmonella* on food. In doing so, we not only identified multiple distinct NTS serovars from two prawn samples, but also some variation in the diversity of the NTS isolates from individual food samples when looking at SNP differences, and some variation in the accessory gene content, specifically AMR genes and plasmid replicons. Overall, 26 % of NTS-positive samples contained isolates from the same serovar that differed by five or more SNPs, and if a single isolate was taken from these samples this variation could hinder the linkage of human cases to potential sources at the five SNP cut-off, should a true epidemiological link exist.

To evaluate if this variation was due to biases in the methods used to enrich and culture NTS, we examined associations between method and the serovars identified. The most effective culturing method for detecting NTS in the samples investigated was using both MKTTn and MSRV enrichments followed by culturing on BSA, but MSRV identified a serovar not identified using MKTTn along with more diverse NTS isolates. However, there may have been *Salmonella* isolates present in the food samples in this study that were missed by all methods. Yangkam Yhiler *et al.* [48] evaluated culturing methods for detecting NTS from chicken, and also found they detected more NTS from chicken by using both MSRV and MKTTn enrichment, but that MSRV was more sensitive. They used XLD and Brilliance Green (BG) for culturing and confirmed NTS using biochemical testing and polyvalent antisera, whilst in this study we used the higher resolution WGS to identify and characterise serovars. Regardless, both studies demonstrate that effective culturing of NTS from food relies on using multiple enrichment methods.

*Salmonella* from food was compared with isolates derived from human cases to determine its potential clinical impact. In England and Wales, 95 % of NTS isolates from human clinical cases identified in diagnostic laboratories are sent to the UKHSA, whole genome sequenced and the genomes made publicly available [17]. We compared the NTS isolates we collected from food to the clinical UK isolates to determine their genetic relatedness. A higher proportion of chicken samples (6.1 %) contained NTS that were within five SNPs of human clinical isolates compared to pork (0.6 %) and raw prawn (0 %) samples. In addition, a significantly higher proportion of imported chicken samples (17 %) were within five SNPs to human isolates compared to domestic chicken samples (2.3 %). Although NTS was more commonly isolated from imported chicken samples [21], it is not clear if this represents a higher food safety risk, as these closely related human isolates were collected up to three years before or a year after those from the food samples. Furthermore, in this study, 85 % of imported chicken samples were frozen and a survey of consumer cooking practices of frozen chicken products in the UK found that only 54 % of consumers checked the cooking instructions [49]. Therefore, the larger risk of imported chicken may in part be due to consumers undertaking unsafe cooking practices with this food commodity, highlighting the importance of considering human behaviour and food consumption practices when evaluating the risks of particular food products [50]. WGS can support epidemiological findings but cannot replace it.

*S.* I 4,[5],12:i-, *S.* Enteritidis and *S.* Infantis were the serovars collected from retail food samples that included isolates that were within five SNPs of clinical cases. *S.* Infantis was the most common serovar isolated in this study, found in domestic and imported chicken samples. *S.* Infantis has increased in prevalence in countries around the world over the last two decades [51, 52], attributed to the pESI plasmid that encodes resistance to antimicrobial agents and increases pathogenicity and biofilm formation [53]. pESI-containing *S.* Infantis emerged in the UK in 2000 and increased in prevalence, with *S.* Infantis now the fifth most common serovar associated with human salmonellosis cases in England and Wales [18]. All *S.* Infantis isolates identified in this study were ST 32 and demonstrated presence of the pESI plasmid [43]. Most of the pESI-containing *S.* Infantis isolates described worldwide are missing region 1 [18], like those described in this study. In 2013, extended-spectrum beta-lactamase (ESBL)-producing *S.* Infantis strains emerged due to the *bla*CTX-M-65 gene. None of the *S.* Infantis isolates in this study contained this gene, but the plasticity of the pESI plasmid could allow them to obtain additional AMR genes in response to antimicrobial selective pressures and expose consumers to more resistant NTS.

The *S.* I 4,[5],12:i- serovar was the only serovar isolated from multiple food commodities in this study; all isolates were ST 34 and were collected from two domestic pork samples and one domestic chicken sample from three different chain supermarkets. *S.* I 4,[5],12:i- carries genes conferring resistance to copper, which is believed to enable its persistence in some settings as copper is used as a growth promoter in pig rearing [54]. *S.* I 4,[5],12:i- has been previously isolated from poultry sources in the UK but not as commonly as from porcine sources [55].

No *S.* Weltevreden isolates collected from food were within five SNPs of human UK sources, suggesting they are not a potential source of outbreaks [17]. However, *S.* Weltevreden from some prawn sources were within ten SNPs of UK human isolates, suggesting they are related to isolates from potential outbreak sources. *S.* Weltevreden is usually associated with aquatic environments and animals that inhabit these environments, especially in South East Asia [56]. All *S.* Weltevreden-positive samples collected in this study were black tiger prawns (*Penaeus monodon*) from Vietnam (*n*=4 samples), Indonesia (*n*=1 sample) or of unknown (*n*=1 sample) origin. *S.* Weltevreden isolates have previously been shown to be less virulent than *S.* Typhimurium isolates, and few contain AMR genes [57], but continue to cause outbreaks worldwide [58]. Global comparisons of *S.* Weltevreden are required to better understand this emerging pathogen.

The number of *S.* Enteritidis cases in the UK has decreased since the 1980s with the introduction of a vaccine against this serovar for hens [59], which likely explains the lack of *S.* Enteritidis from domestic chicken samples in this study. However, *S.* Enteritidis is still a major cause of salmonellosis in the UK [45]; recently, *S.* Enteritidis from imported breaded chicken was associated with over 100 human cases in the UK [60] and a subsequent investigation of frozen chicken in the UK found they belonged to ST 11 and linked them to chicken products produced in Poland [19]. The chicken samples that contained *S.* Enteritidis in this study were also all of Polish origin or originated from multiple countries including Poland, but belonged to ST 4747 and two novel STs, suggesting that imported chicken is exposing consumers to *S.* Enteritidis with STs other than ST 11.

Ten other *Salmonella* serovars were isolated from the food commodities examined in this study. Although no isolates were within five SNPs of publicly available *Salmonella* genomes from clinical cases, it is possible that isolates belonging to these serovars could acquire genetic factors that would allow them to colonise animal hosts more effectively, and therefore increase in prevalence, as was the case with the *S.* Infantis serovar and acquisition of the pESI plasmid [18, 51, 52]. Continued surveillance of retail food, which is the point closest to the consumer, is required to monitor the serovars in the food chain and WGS is required to determine the likelihood that isolates belonging to these serovars could be contributing to clinical cases.

Identifying where NTS contaminates foods such as chicken, pork and prawns is challenging, as it can derive from colonisation of the animal itself [61], or from cross-contamination that can occur during slaughtering, processing and handling points [62, 63]. NTS genomes have a substitution rate of 1.5–4.5×10^−7^ substitutions per site per year [14, 15, 64], which equates to one or two SNPs per genome per year. Broiler chickens have a lifespan of approximately five weeks [65], pigs for slaughter a lifespan of approximately 28 weeks [66] and aquacultured prawns a lifespan of 28–33 weeks [67]. If these animals were colonised with a single NTS at the beginning of their lifespans it would not be expected for the NTS to accumulate more than two SNPs before the exposure point to humans directly through food. In total, 64 % of the 42 NTS-positive samples in this study contained isolates that shared two or fewer SNPs; the rest of the samples were either colonised with a genetically diverse NTS population or may have been contaminated at multiple points in the production chain with diverse NTS. This population diversity highlights the need to culture multiple NTS isolates from food samples.

In this study we investigated the genetic diversity of NTS from retail food sold in Norfolk, UK, and assessed the risk of infection by NTS based on how distantly related they were to clinical cases from the UK, the number of AMR determinants they contained and the diversity of isolates found on samples. Overall, the prevalence of *Salmonella* contamination on these food products was low, as previously reported [21], but the application of WGS revealed genetic diversity of *Salmonella* on these foods, which may hinder outbreak investigations and source attribution. It is important to note that given the dynamic and shifting nature of food sources, farming and production practices and consumer behaviour, the types of foods associated with foodborne disease may change over time and by geographical location. Therefore, the prevention of future salmonellosis cases and outbreaks relies on continued surveillance of NTS found on retail food, with the high resolution offered by WGS allowing greater insight into its relationship to human clinical cases.

## Supplementary Data

Supplementary material 1
