# Supplementary material for: Genomic diversity and epidemiological significance of non-typhoidal Salmonella found in retail food collected in Norfolk, UK
Source: Microb Genom. 2023 Jul 31;9(7):mgen001075. doi: 10.1099/mgen.0.001075 (PMC10438825; doi:10.1099/mgen.0.001075)
Supplement: Supplementary material 1 [file mgen-9-1075-s001.pdf]

Supplementary material

Supplementary table 1. Metadata of the food samples that cultured for *Salmonella*

Sample	Year	Store type	Commodity	Product presentation	Country source
**CH-0043**	2018	Chain	Chicken	Frozen	Imported
**CH-0053**	2018	Chain	Chicken	Frozen	Imported
**CH-0060**	2018	Chain	Chicken	Chilled	Imported
**CH-0062**	2018	Chain	Chicken	Frozen	Imported
**CH-0070**	2018	Chain	Chicken	Frozen	Imported
**CH-0073**	2018	Chain	Chicken	Frozen	Imported
**CH-0087**	2018	Chain	Chicken	Chilled	Domestic
**CH-0089**	2018	Chain	Chicken	Frozen	Imported
**CH-0105**	2018	Chain	Chicken	Frozen	Imported
**CH-0106**	2018	Butcher	Chicken	Chilled	Domestic
**CH-0111**	2018	Chain	Chicken	Frozen	Imported
**CH-0117**	2019	Chain	Chicken	Frozen	Imported
**CH-0126**	2019	Chain	Chicken	Chilled	Domestic
**CH-0127**	2019	Chain	Chicken	Frozen	Imported
**CH-0134**	2019	Chain	Chicken	Chilled	Imported
**CH-0138**	2019	Butcher	Chicken	Chilled	Domestic
**CH-0146**	2019	Chain	Chicken	Chilled	Domestic
**CH-0147**	2019	Chain	Chicken	Frozen	Imported
**CH-0150**	2019	Chain	Chicken	Chilled	Imported
**CH-0163**	2019	Chain	Chicken	Frozen	Imported
**CH-0180**	2019	Chain	Chicken	Frozen	Imported
**CH-0240**	2019	Chain	Chicken	Frozen	Imported
**CH-0246**	2019	Chain	Chicken	Chilled	Domestic
**CH-0255**	2019	Chain	Chicken	Frozen	Imported
**CH-0261**	2019	Butcher	Chicken	Chilled	Domestic
**CH-0265**	2019	Chain	Chicken	Chilled	Domestic
**CH-0273**	2019	Chain	Chicken	Chilled	Domestic
**CH-0278**	2019	Chain	Chicken	Frozen	Imported
**CH-0304**	2019	Chain	Chicken	Frozen	Unknown
**CH-0311**	2019	Chain	Chicken	Frozen	Imported
**PK-0042**	2018	Chain	Pork	Chilled	Domestic
**PK-0137**	2019	Chain	Pork	Chilled	Domestic
**PK-0242**	2019	Butcher	Pork	Chilled	Domestic
**PK-0299**	2019	Chain	Pork	Chilled	Domestic
**PR-0007**	2018	Chain	Prawns	Frozen	Imported
**PR-0037**	2018	Chain	Prawns	Frozen	Imported
**PR-0067**	2018	Chain	Prawns	Chilled	Unknown
**PR-0126**	2018	Independent	Prawns	Chilled	Unknown
**PR-0151**	2018	Chain	Prawns	Frozen	Imported

**PR-0191**	2019	Chain	Prawns	Chilled	Imported
**PR-0198**	2019	Chain	Prawns	Frozen	Imported
**PR-0238**	2019	Chain	Prawns	Frozen	Imported

Read trimming

Raw paired-end reads were trimmed using the following Trimmomatic command:

trimmomatic PE -threads <number of threads> -phred33 <forward read file> < reverse read file> <forward paired read outfile> <forward unpaired read outfile> <reverse paired read outfile> <reverse unpaired read outfile> ILLUMINACLIP:<library adapter file>:2:30:10 LEADING:3 TRAILING:3 SLIDINGWINDOW:4:25 MINLEN:50

Serovars

Some of the NTS serovars isolated from chicken samples were associated with country of origin (Supplementary figure 1): *S.* Newport (*n* = 2 samples) and *S.* Enteritidis (*n* = 9 samples) serovars were only isolated from imported samples, whilst *S.* I,4,[5],12:i:- (*n* = 1 sample), *S.* Kedougou (*n* = 1 sample), *S.* Mbandaka (*n* = 1 sample) and *S.* Ohio (*n* = 2 samples) serovars were only isolated from domestic samples. The *S.* Infantis (*n* = 14 samples) serovar was isolated from domestic chicken samples (*n* = 4 samples), imported chicken samples (*n* = 9 samples), and a chicken sample whose origin was unknown (*n* = 1 sample). All the chicken NTS isolates were obtained from chain supermarkets, apart from three samples from butchers from which *S.* Infantis was isolated.

There was also an association between the serovar isolated from chicken samples and how they were presented. *S.* I,4,[5],12:i:- (*n* = 1 sample), *S.* Kendougou (*n* = 1 sample), *S.* Mbandaka (*n* = 1 sample), *S.* Ohio (*n* = 2 sample) were only found in chilled chicken

samples, whilst *S.* Newport (n = 2 samples) were only isolated from frozen chicken samples. *S.* Enteritidis and *S.* Infantis serovars were isolated from both chilled (n = 1 sample and n = 6 samples, respectively) and frozen (n = 8 samples and n = 8 samples, respectively). However, these associations are likely confounded by associations between the origin of chicken samples and how they were presented (stored).

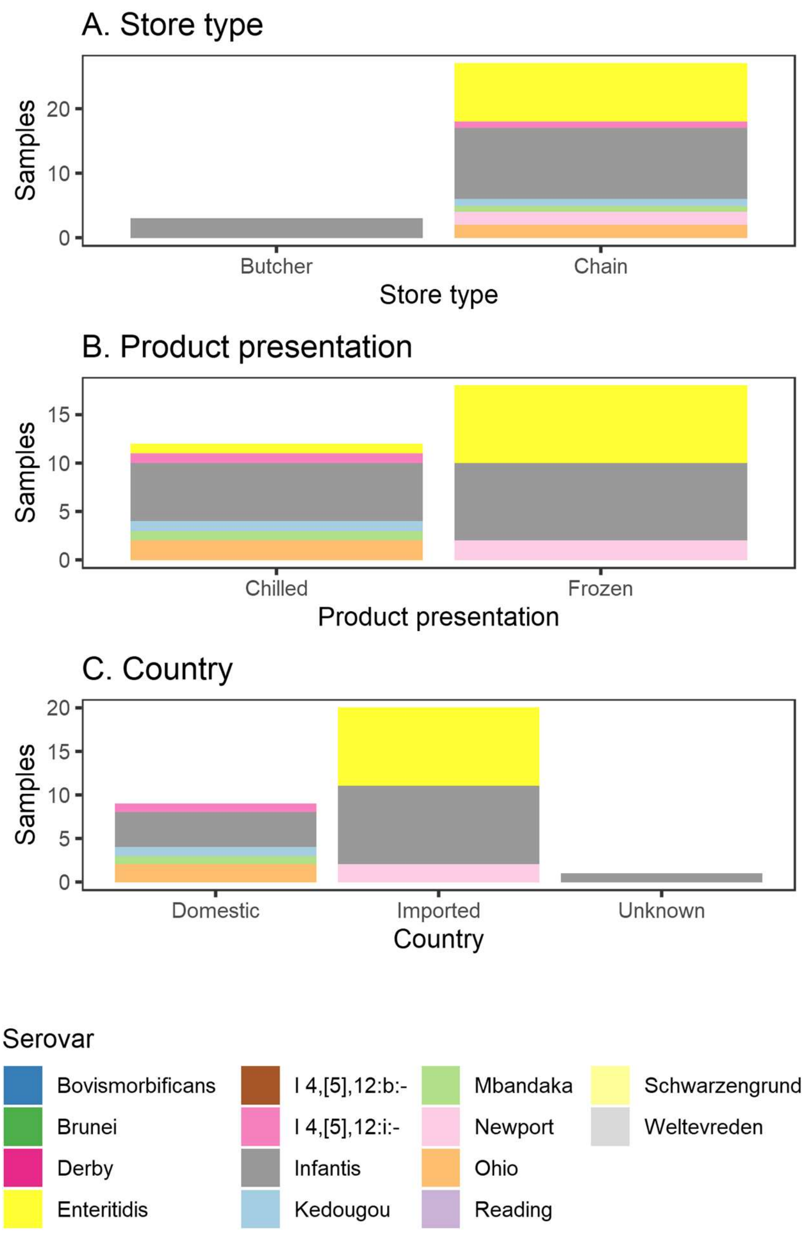

Supplementary figure 1. Bar graphs of the number of chicken samples from different store types (A), product presentations (B) and countries (C) and coloured by the NTS serovar identified.

AMR determinants

For chicken samples that tested positive for NTS, all (3/3) butcher samples contained MDR NTS isolates compared to 48% (13/27) of chain supermarket samples, but these percentages were not significant ($p = 0.099$) (Supplementary figure 2). In terms of storage, 58% (7/12) of chilled samples contained MDR NTS compared to 50% (9/18) of frozen samples, and these percentages were not significant ($p = 0.66$). In terms of country of origin, 50% (10/20) of imported samples contained MDR NTS isolates, compared to 56% (5/9) of imported samples and these were not significant ($p = 0.78$). All NTS isolates collected from chicken of unknown origin were MDR, but they were all isolated from one sample..

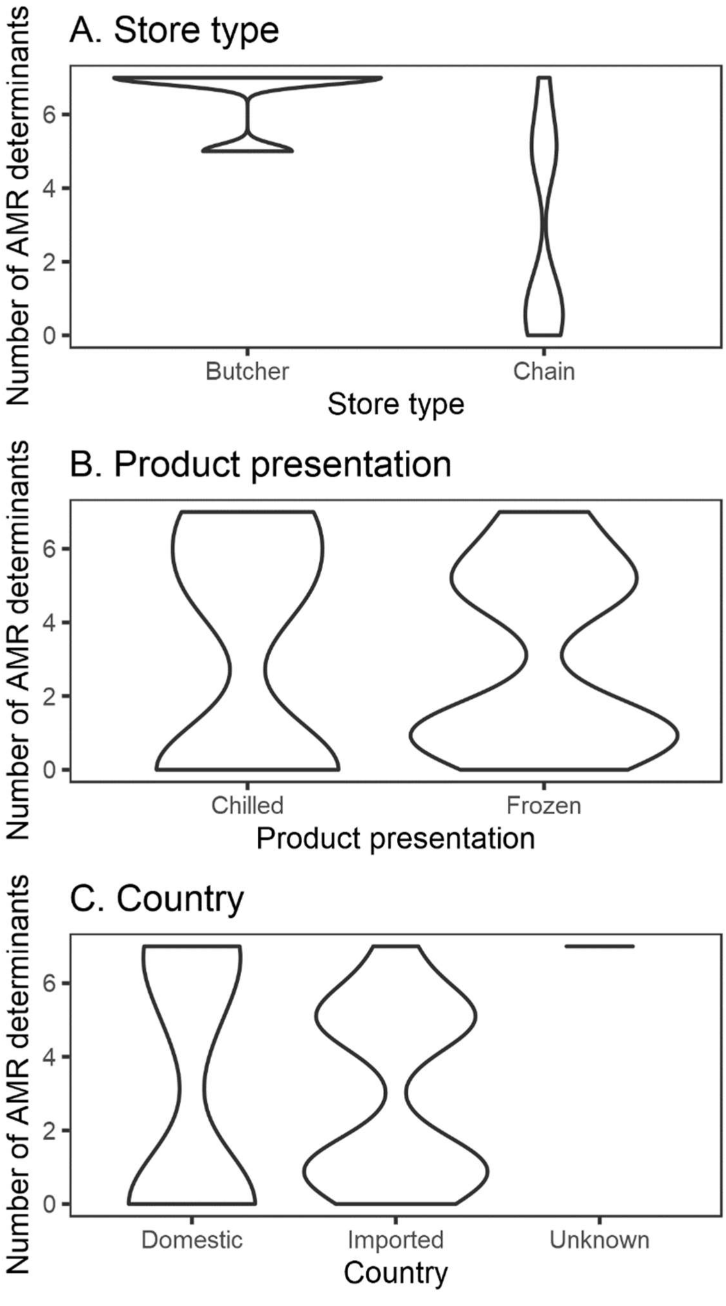

Supplementary figure 2. Violin plots of the number of AMR determinants identified in NTS from chicken samples from different store types (A), product presentations (B) and countries (C).

pESI plasmid coverage

For all the NTS isolates collected from food samples, the read coverage to the pESI plasmid was calculated (Supplementary figure 3). Only the isolates belonging to the *S.* Infantis serovar had plasmid coverages above 70%.

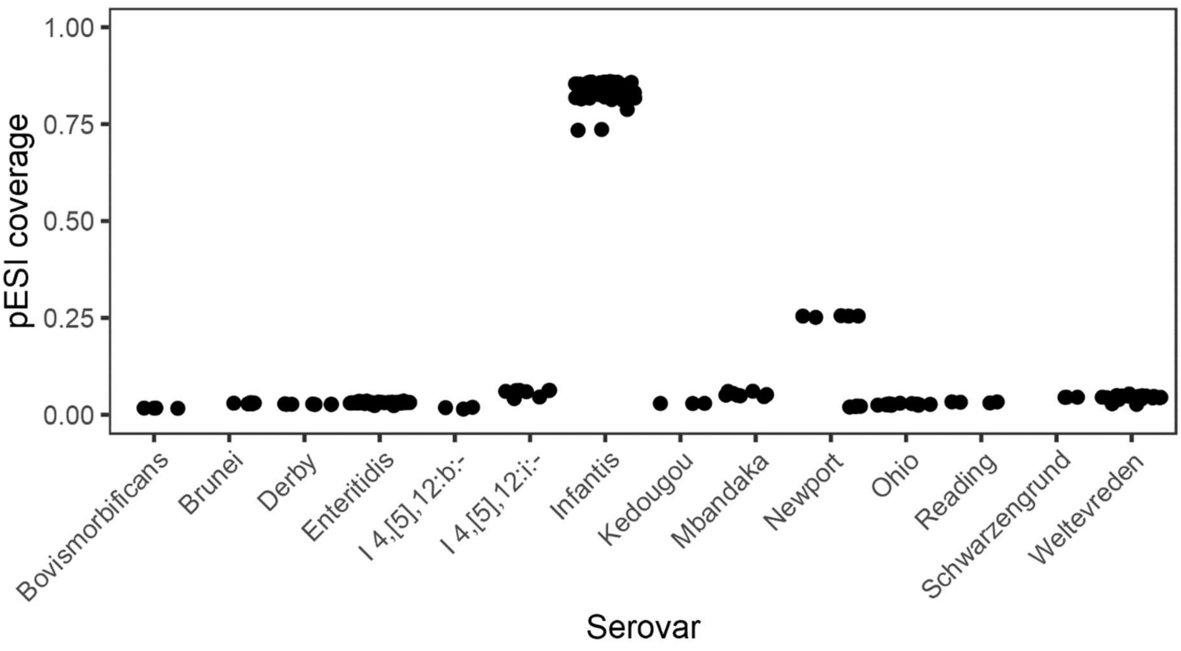

Supplementary figure 3. Jitter plot of the read coverage to the pESI plasmid for NTS isolates separated by serovar.

Method diversity comparison

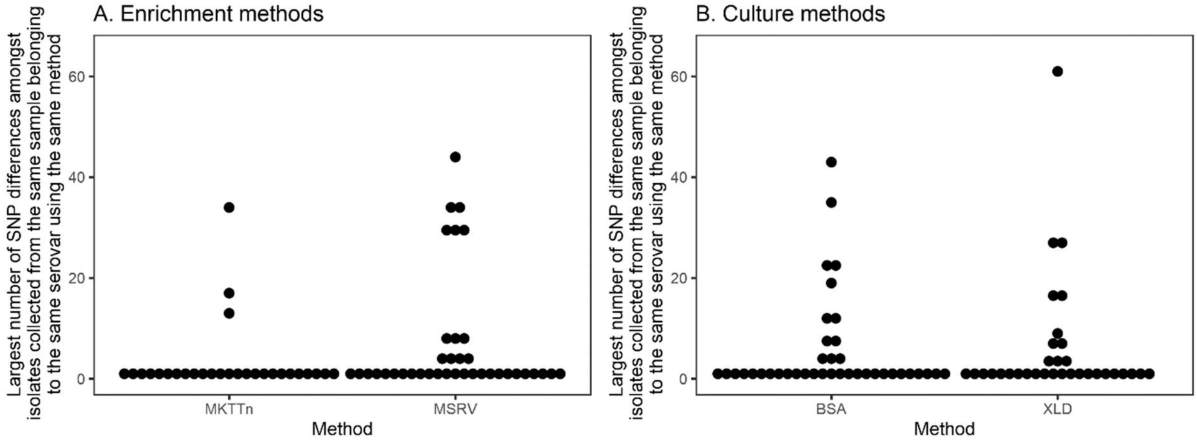

Supplementary figure 4. Dot plots of the largest number of SNP differences amongst isolates collected from the same sample belonging to the same serovar using enrichment (A) and culture (B) methods.

Comparison with human isolates

The *Salmonella* isolates collected as part of this study (food surveillance isolates) were compared to publicly available genomes on EnteroBase using hierarchical clustering. However, the number of publicly available genomes differed for each serovar, resulting in different levels of hierarchical clustering used for each serovar (Supplementary table 2).

Supplementary table 2. Hierarchical clustering levels, references and the number of isolates compared for each serovar

Serovar	Hierarchical clustering level	Food surveillance isolates	Human isolates	Non-human isolates	Reference
**Bovismorbificans**	100	4	22	0	SAMEA3138815
**Brunei**	400	5	6	14	SAMEA4557834*
**Derby**	50	8	29	6	SAMN06045055
**Enteritidis**	10	42	1456	31	SAMN06050736
**Infantis**	100	66	1104	62	SAMN11355817
**I 4,[5],12:b:-**	900	3	352	8	SAMEA3506109*
**I 4,[5],12:i:-**	100	9	2123	223	SAMN11333171
**Kedougou**	50	3	107	16	SAMN14832905
**Mbandaka**	100	8	278	41	SAMN06044996
**Newport**	400	9	257	4	SAMN03744302
**Ohio**	50	13	48	4	SAMN09237646
**Reading**	400	4	3	0	SAMN06044994
**Schwarzengrund**	50	3	15	4	SAMN13031563
**Weltevreden**	200	22	303	23	SAMEA1904401

*No references available from these serovars, so references from other serovar used

Maximum likelihood trees were formed for each of the serovars, comparing the food surveillance NTS isolates to the most closely related isolates in the United Kingdom from that serovar (Supplementary figures 5-18).

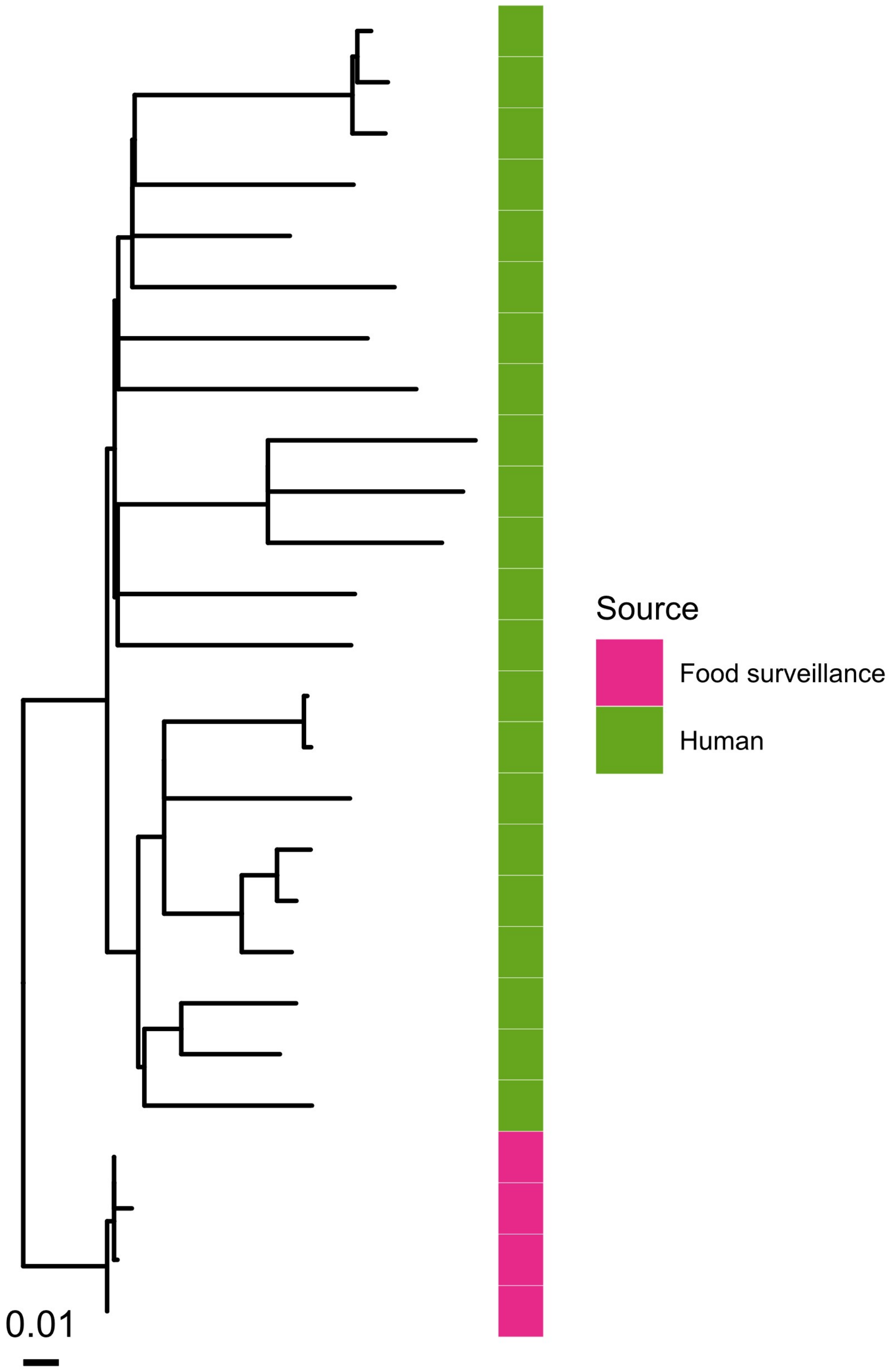

Supplementary figure 5. Maximum likelihood tree of *S.* Bovismorbificans isolates collected from the food surveillance study and the most closely related genomes from the United Kingdom and coloured by source. The phylogenetic branch lengths are given in nucleotide substitutions per site, therefore a branch of length 0.01 (as represented by the scale bar) equates to 11 substitutions, given that 1,063 SNPs was used to estimate it.

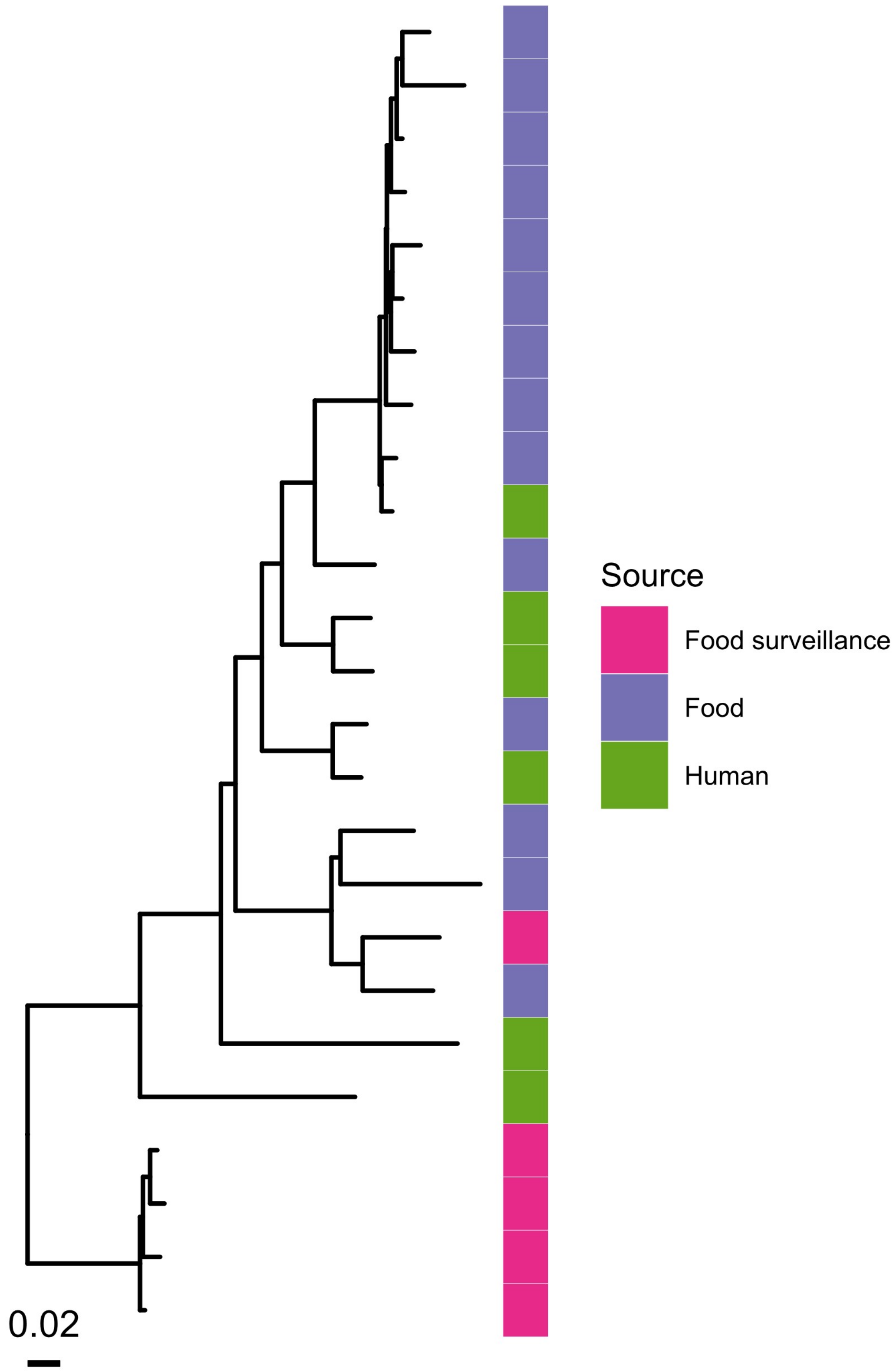

Supplementary figure 6. Maximum likelihood tree of *S.* Brunei isolates collected from the food surveillance study and the most closely related genomes from the United Kingdom and coloured by source. The phylogenetic branch lengths are given in nucleotide substitutions per site, therefore a branch of length 0.02 (as represented by the scale bar) equates to 51 substitutions, given that 2,527 SNPs was used to estimate it.

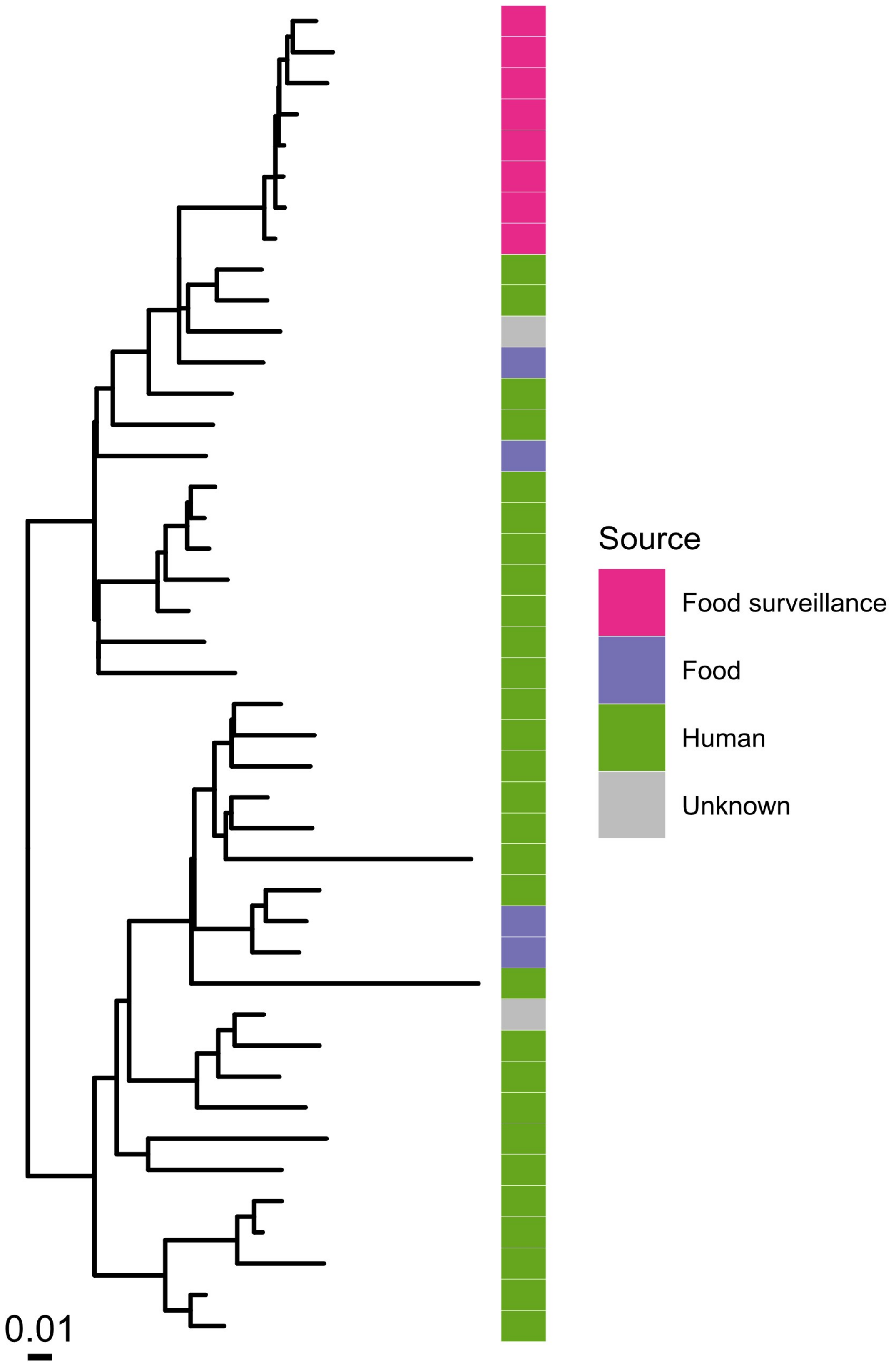

Supplementary figure 7. Maximum likelihood tree of *S.* Derby isolates collected from the food surveillance study and the most closely related genomes from the United Kingdom and coloured by source. The phylogenetic branch lengths are given in nucleotide substitutions per site, therefore a branch of length 0.01 (as represented by the scale bar) equates to 14 substitutions, given that 1,398 SNPs was used to estimate it.

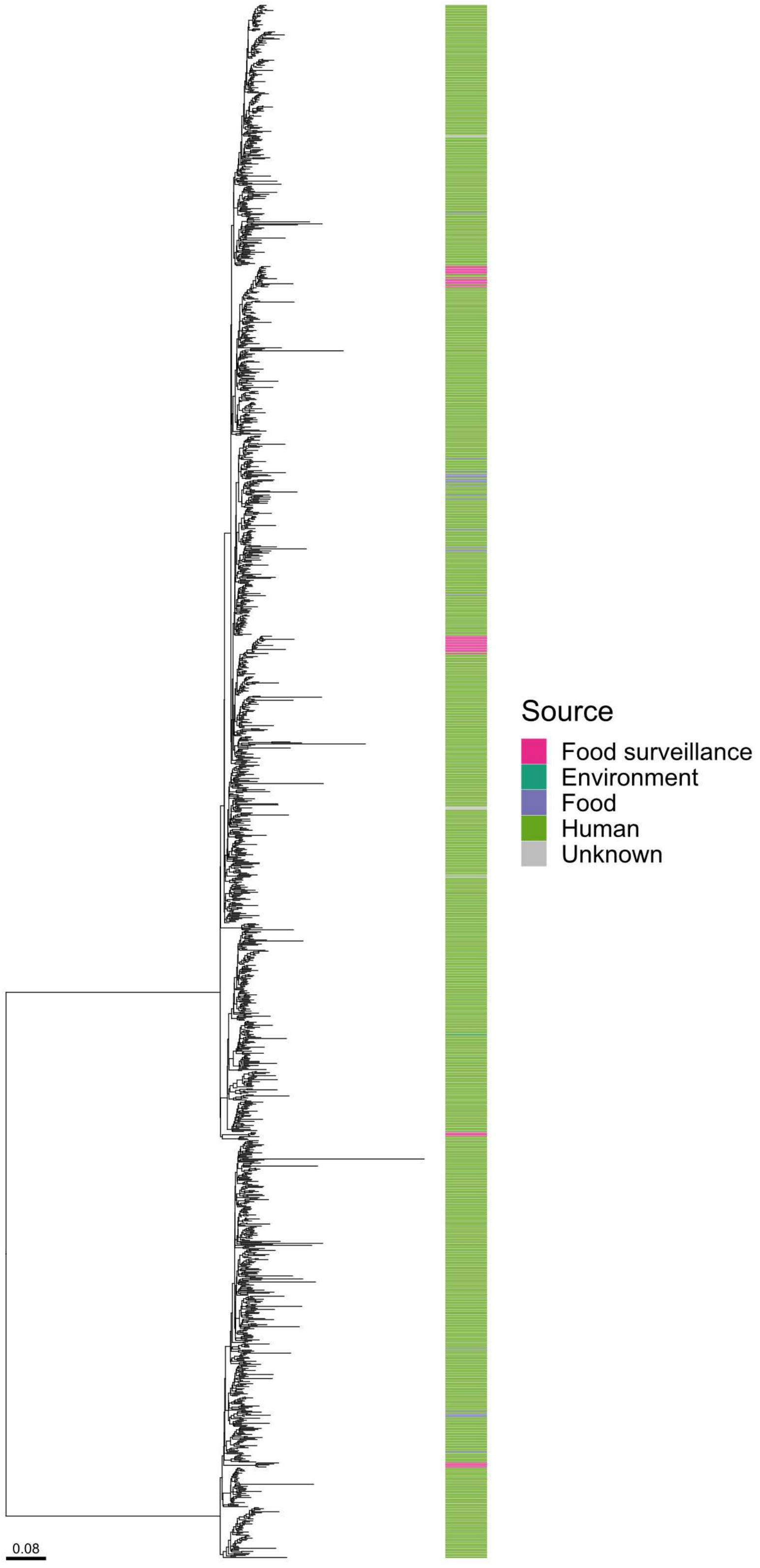

Supplementary figure 8. Maximum likelihood tree of *S.* Enteritidis isolates collected from the food surveillance study and the most closely related genomes from the United Kingdom; coloured by source. The phylogenetic branch lengths are given in nucleotide substitutions per site, therefore a branch of length 0.08 (as represented by the scale bar) equates to 2,975 substitutions, given that 37,188 SNPs was used to estimate it.

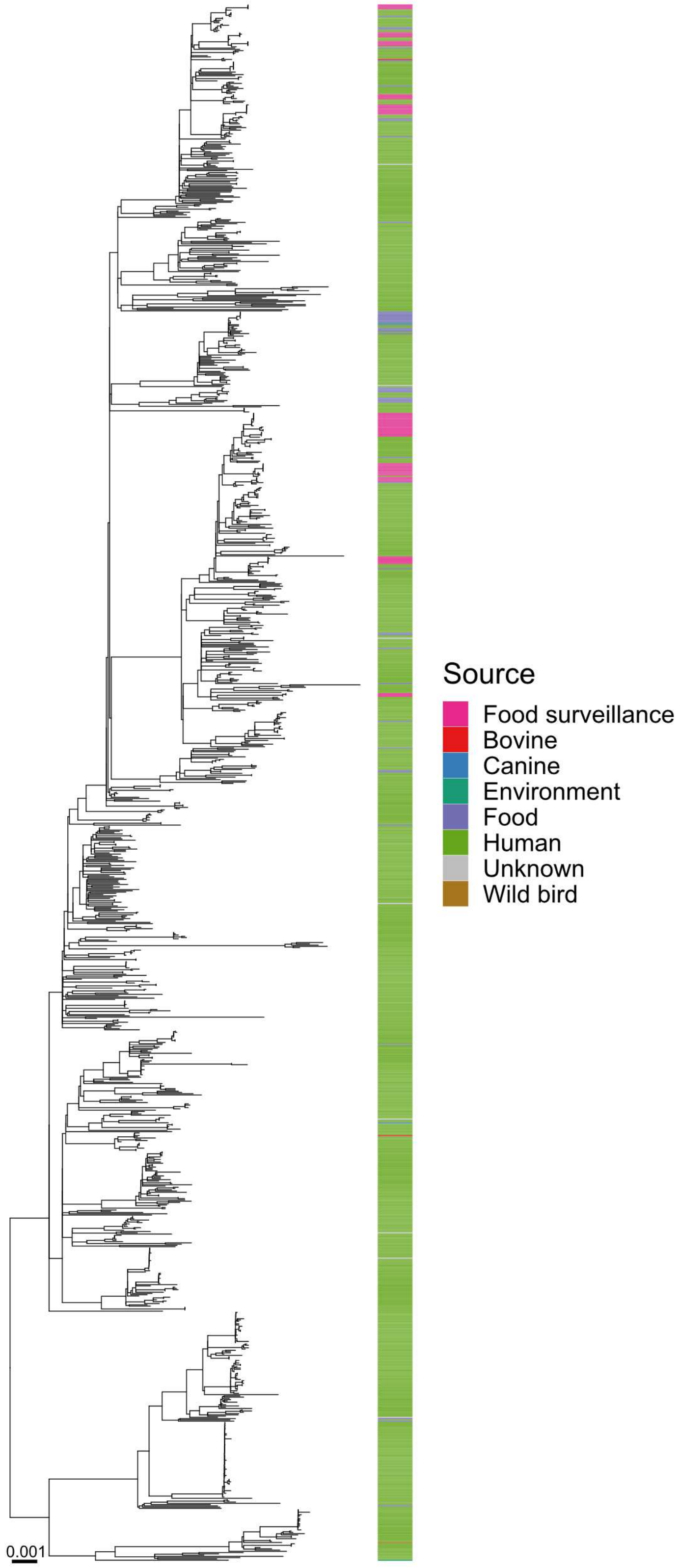

Supplementary figure 9. Maximum likelihood tree of *S.* Infantis isolates collected from the food surveillance study and the most closely related genomes from the United Kingdom and coloured by source. The phylogenetic branch lengths are given in nucleotide substitutions per site, therefore a branch of length of 0.001 (as represented by the scale bar) equates to 16 substitutions, given that 16,379 SNPs was used to estimate it.

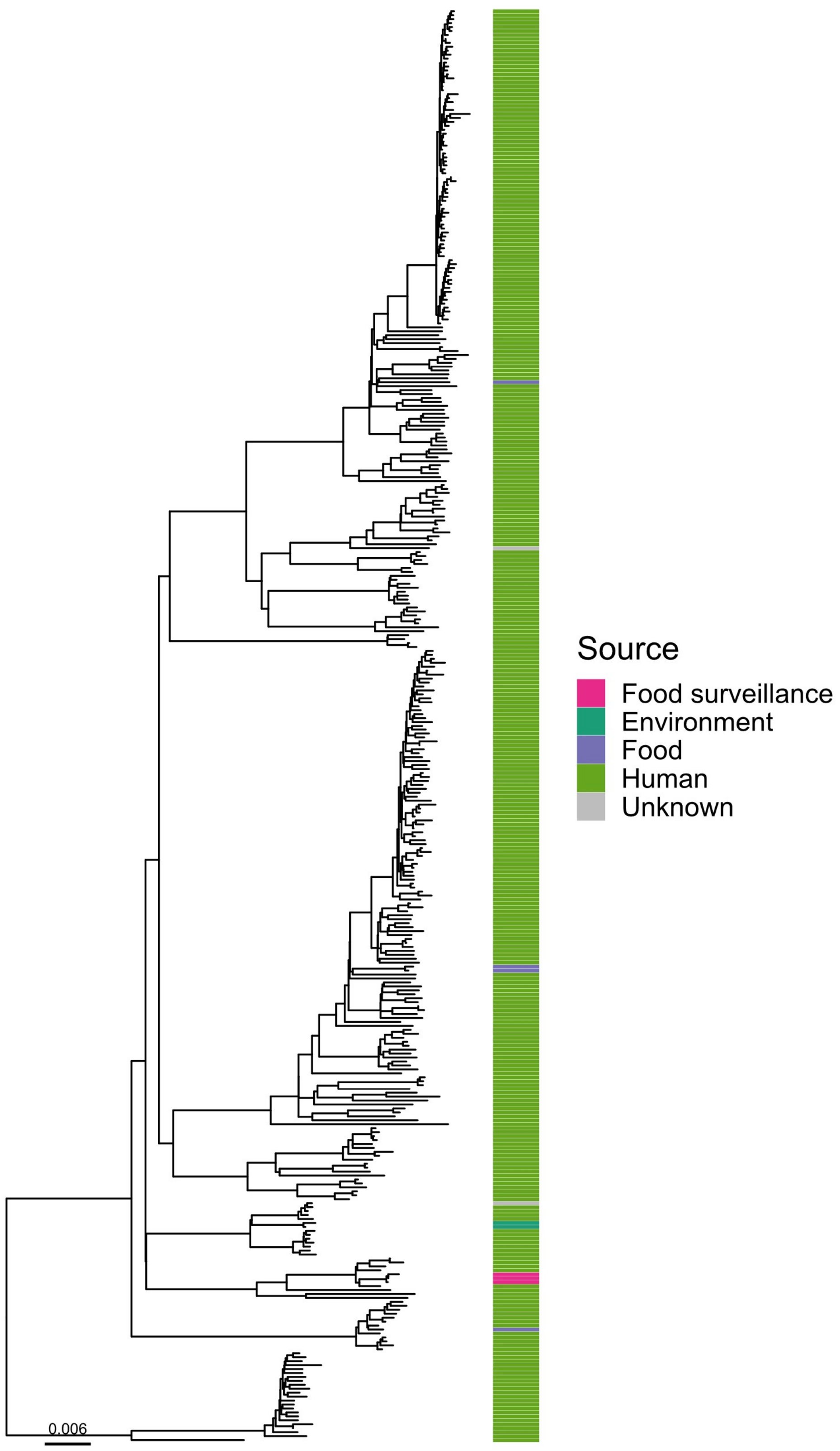

Supplementary figure 10. Maximum likelihood tree of *S.* I 4,[5],12:b:- isolates collected from the food surveillance study and the most closely related genomes from the United Kingdom and coloured by source. The phylogenetic branch lengths are given in nucleotide substitutions per site, therefore a branch of length 0.006 (as represented by the scale bar) equates to 139 substitutions, given that 23,117 SNPs was used to estimate it.

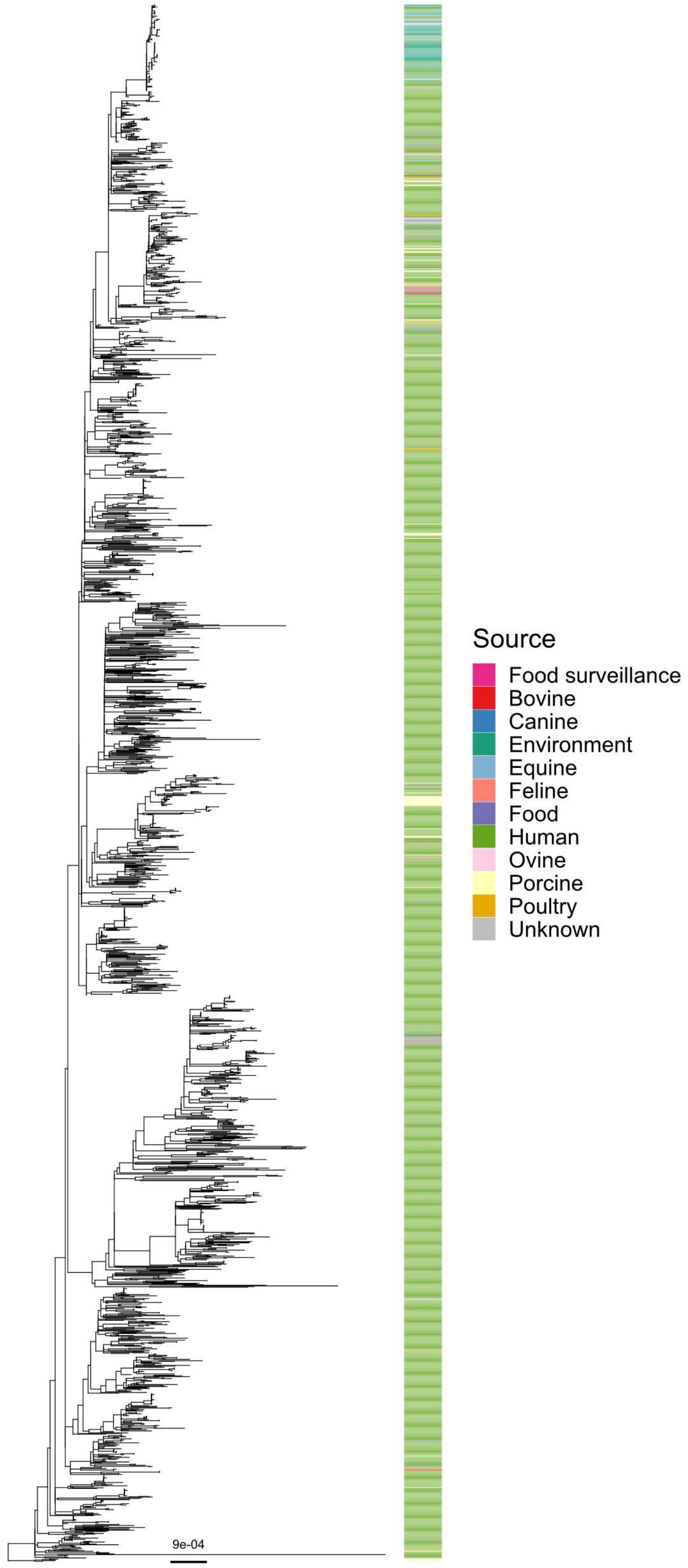

Supplementary figure 11. Maximum likelihood tree of *S.* I 4,[5],12:i:- isolates collected from the food surveillance study and the most closely related genomes from the United Kingdom and coloured by source. The phylogenetic branch lengths are given in nucleotide substitutions per site, therefore a branch of length 0.0009 (as represented by the scale bar) equates to 14 substitutions, given that 16,045 SNPs was used to estimate it.

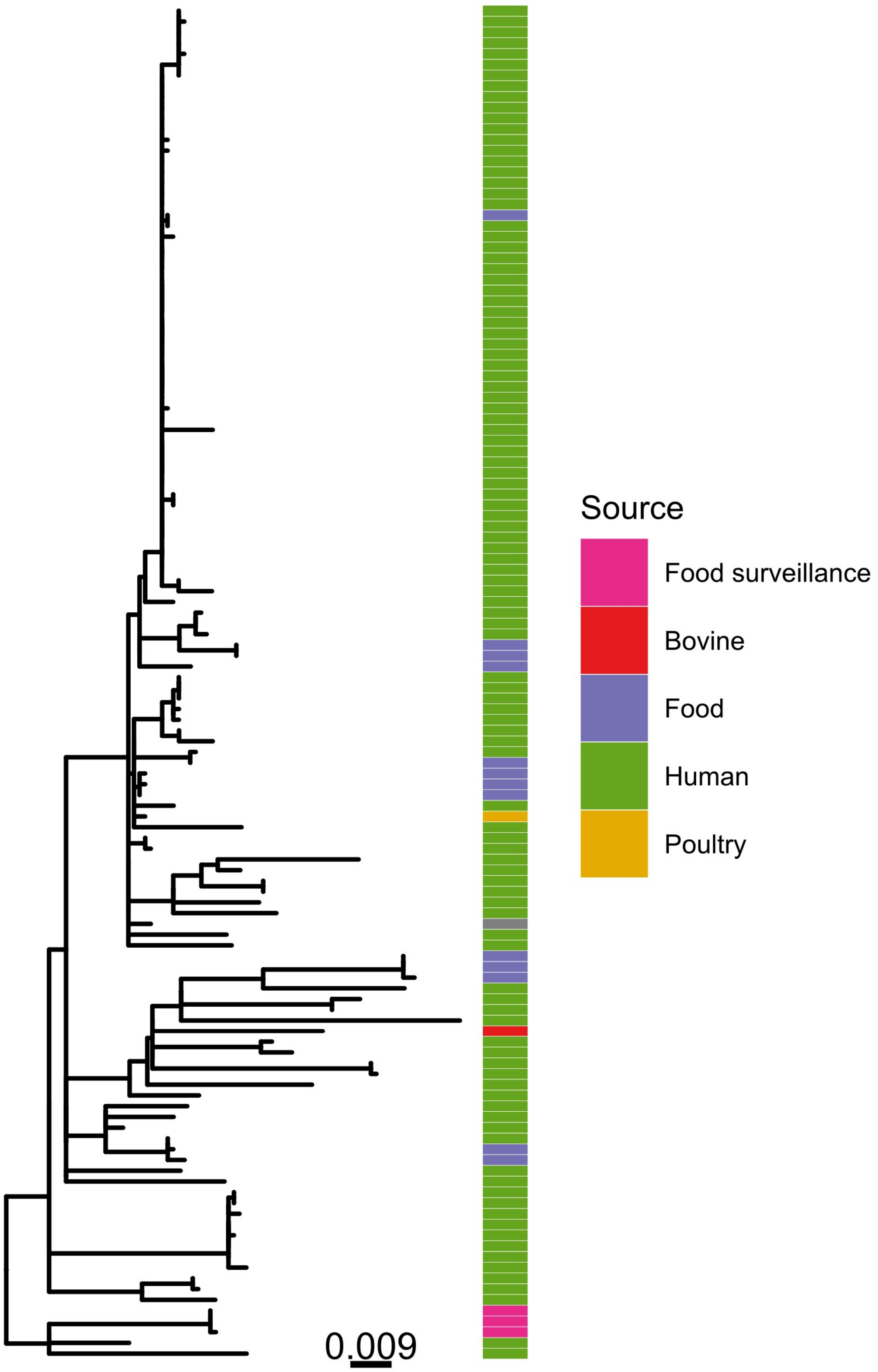

Supplementary figure 12. Maximum likelihood tree of *S.* Kedougou isolates collected from the food surveillance study and the most closely related genomes from the United Kingdom and coloured by source. The phylogenetic branch lengths are given in nucleotide substitutions per site, therefore a branch of length 0.009 (as represented by the scale bar) equates to 7 substitutions, given that 824 SNPs was used to estimate it.

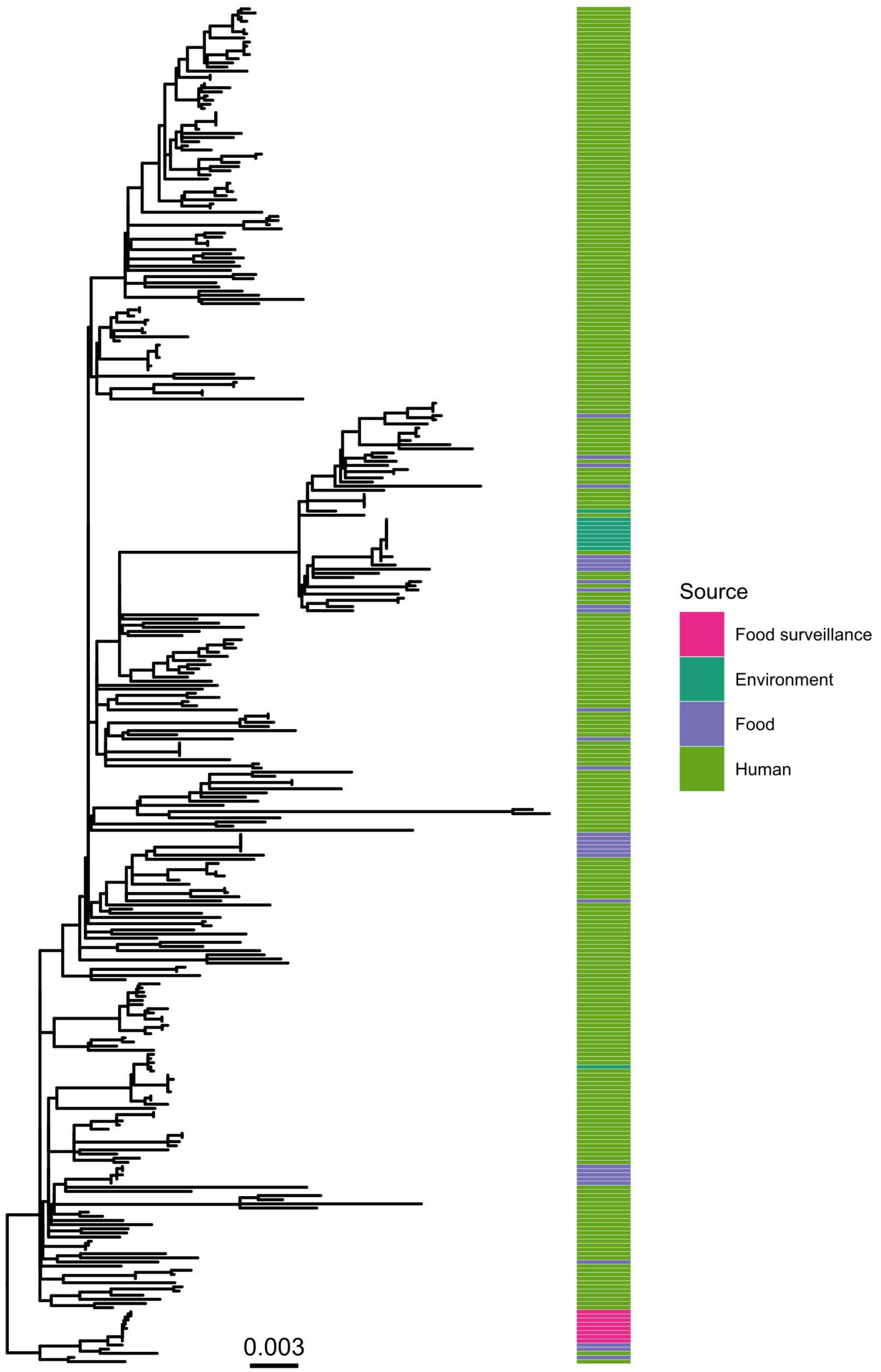

Supplementary figure 13. Maximum likelihood tree of *S.* Mbandaka isolates collected from the food surveillance study and the most closely related genomes from the United Kingdom and coloured by source. The phylogenetic branch lengths are given in nucleotide substitutions per site, therefore a branch of length 0.003 (as represented by the scale bar) equates to 17 substitutions, given that 5,737 SNPs was used to estimate it.

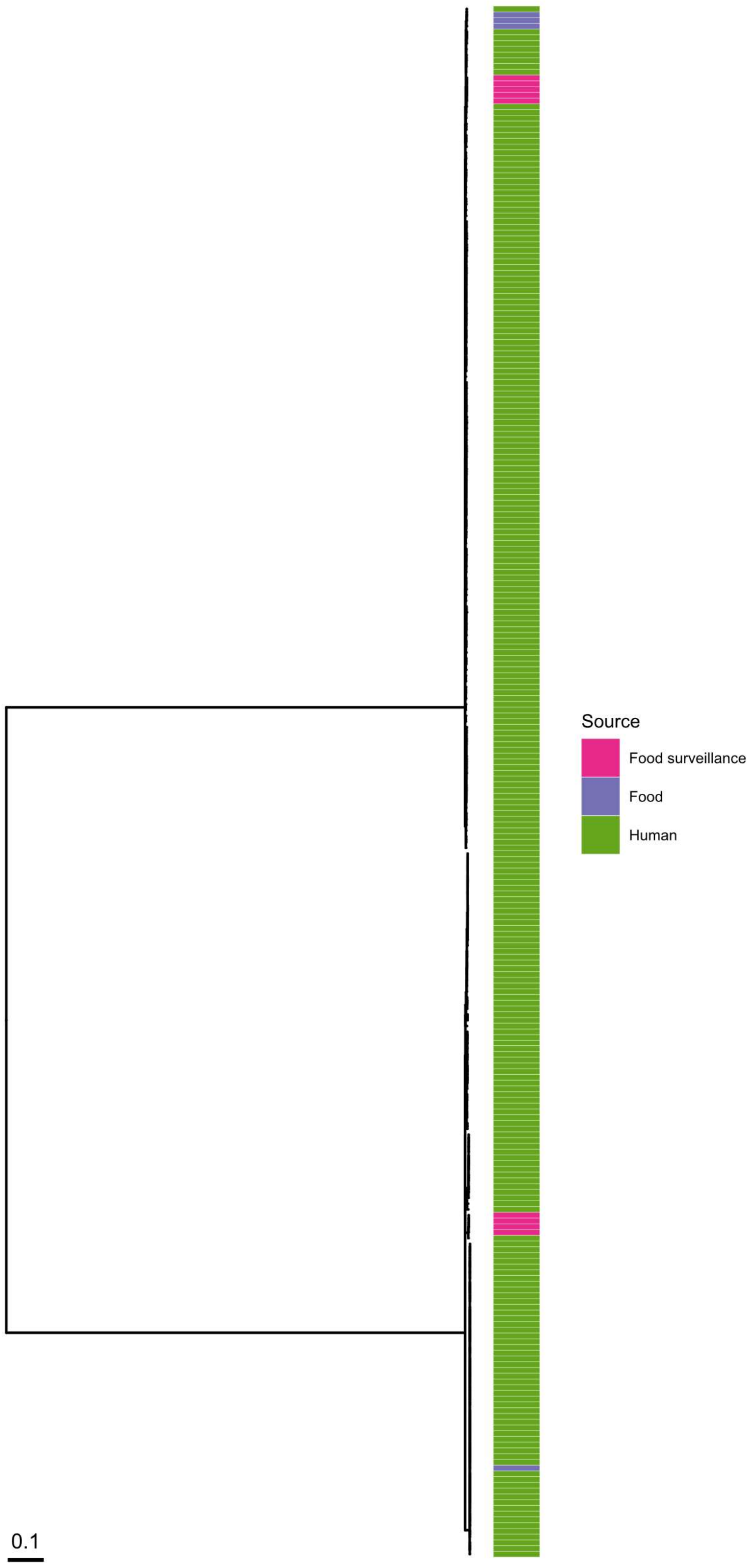

Supplementary figure 14. Maximum likelihood tree of *S.* Newport isolates collected from the food surveillance study and the most closely related genomes from the United Kingdom and coloured by source. The phylogenetic branch lengths are given in nucleotide substitutions per site, therefore a branch of length 0.1 (as represented by the scale bar) equates to 3,269 substitutions, given that 32,690 SNPs was used to estimate it.

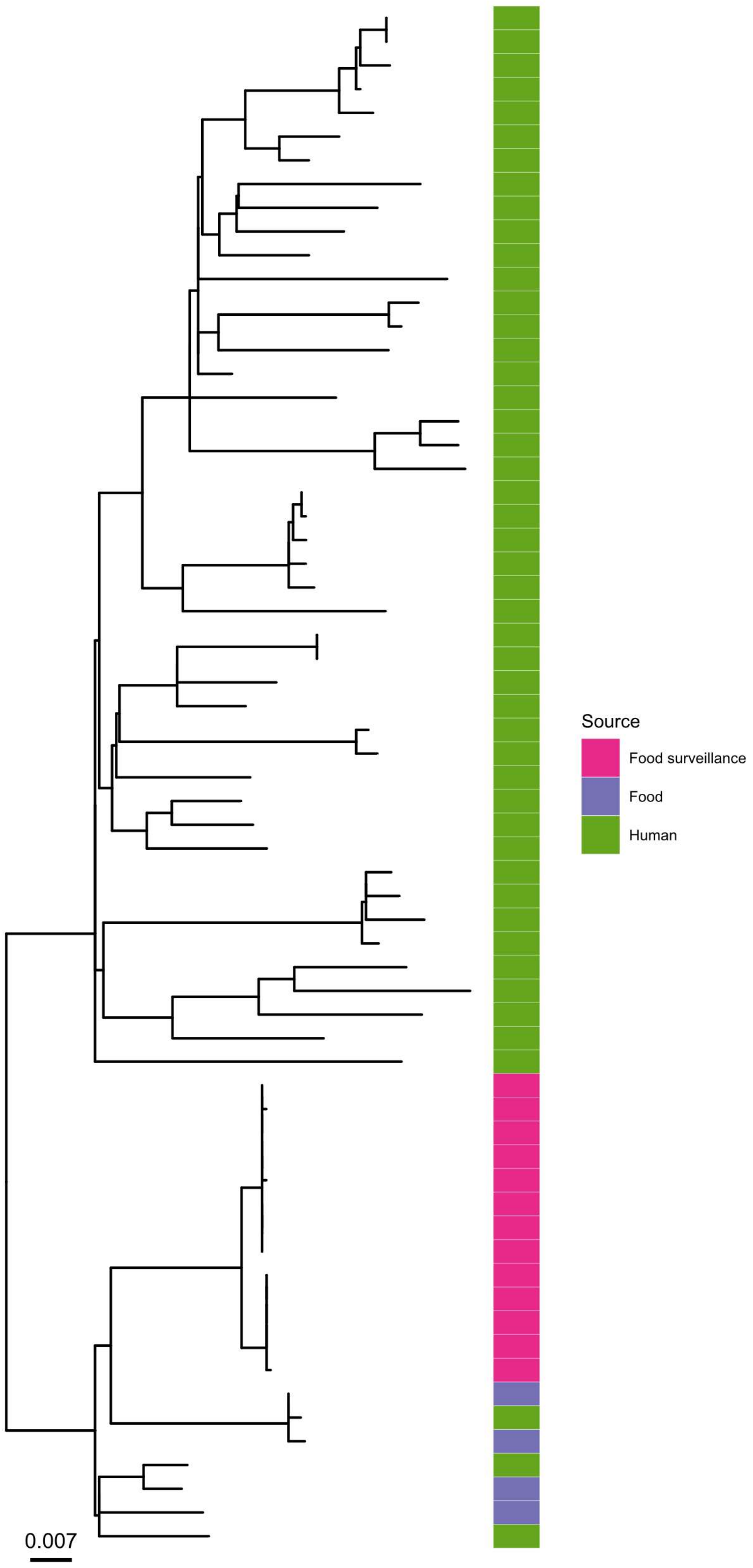

Supplementary figure 15. Maximum likelihood tree of *S.* Ohio isolates collected from the food surveillance study and the most closely related genomes from the United Kingdom and coloured by source. The phylogenetic branch lengths are given in nucleotide substitutions per site, therefore a branch of length 0.007 (as represented by the scale bar) equates to 10 substitutions, given that 1,440 SNPs was used to estimate it.

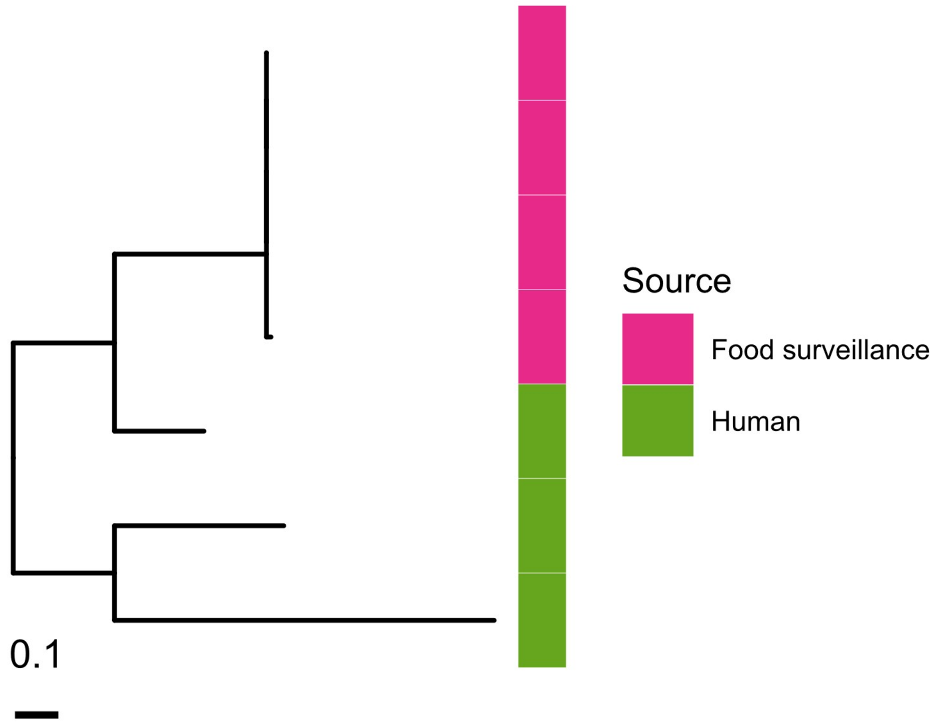

Supplementary figure 16. Maximum likelihood tree of *S.* Reading isolates collected from the food surveillance study and the most closely related genomes from the United Kingdom and coloured by source. The phylogenetic branch lengths are given in nucleotide substitutions per site, therefore a branch of length 0.1 (as represented by the scale bar) equates to 39 substitutions, given that 388 SNPs was used to estimate it.

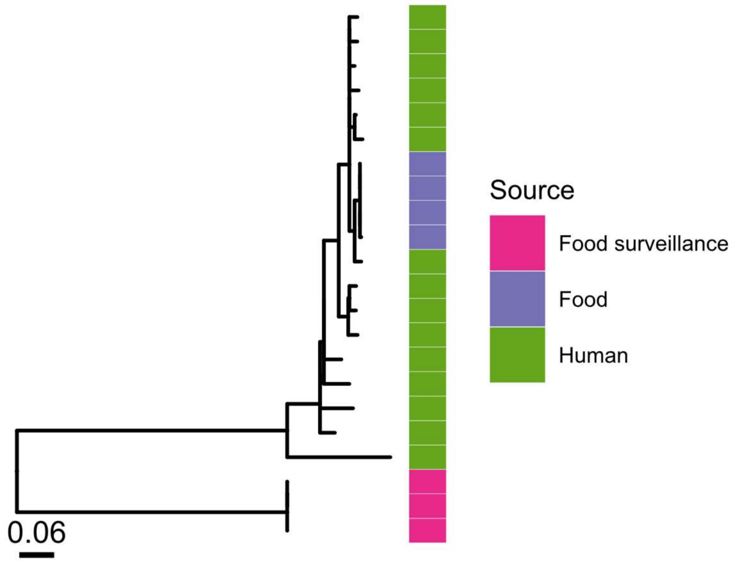

Supplementary figure 17. Maximum likelihood tree of *S.* Schwarzengrund isolates collected from the food surveillance study and the most closely related genomes from the United Kingdom and coloured by source. The phylogenetic branch lengths are given in nucleotide substitutions per site, therefore a branch of length 0.06 (as represented by the scale bar) equates to 27 substitutions, given that 446 SNPs was used to estimate it.

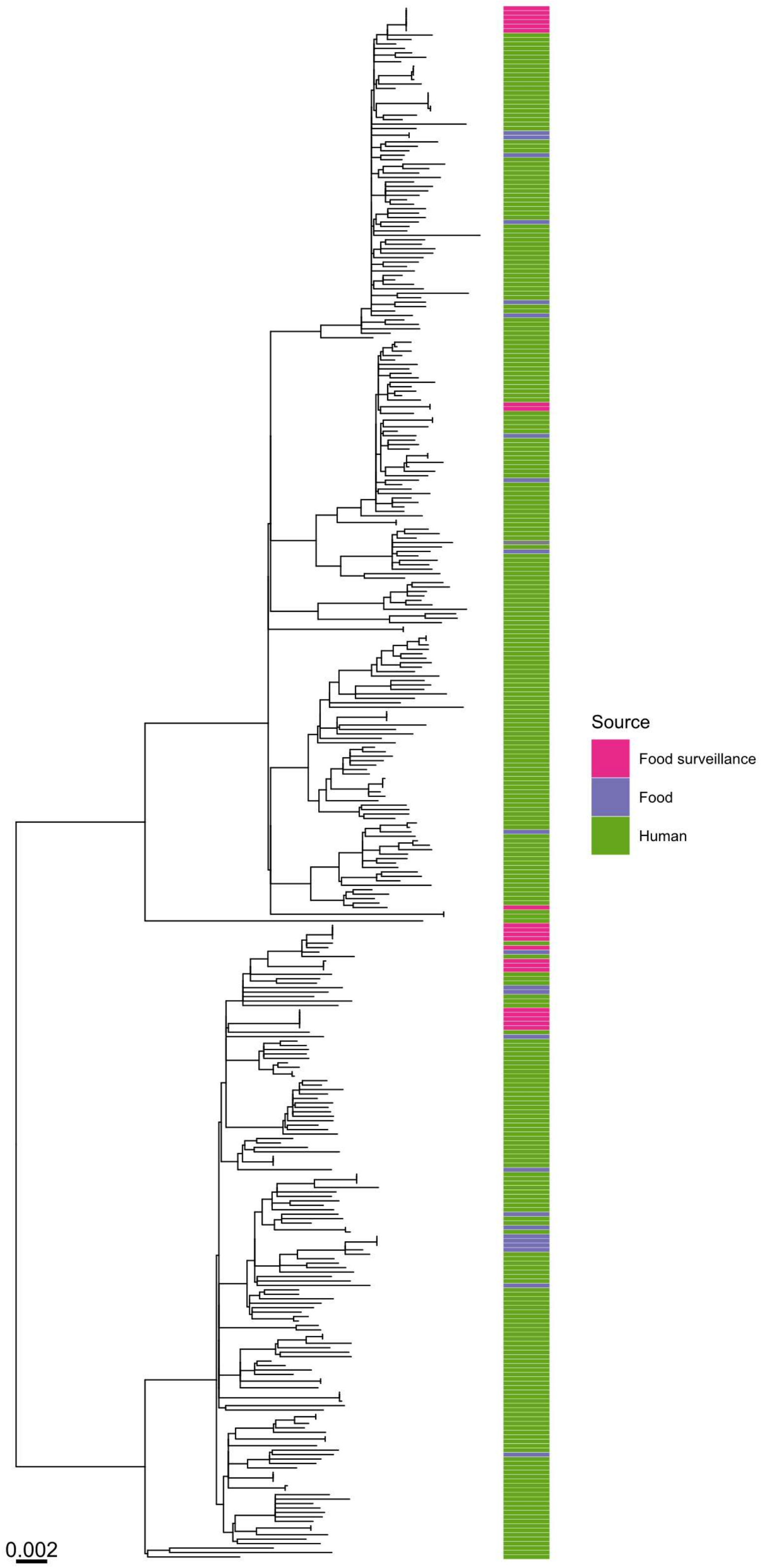

Supplementary figure 18. Maximum likelihood tree of *S.* Weltevreden isolates collected from the food surveillance study and the most closely related genomes from the United Kingdom and coloured by source. The phylogenetic branch lengths are given in nucleotide substitutions per site, therefore a branch of length 0.002 (as represented by the scale bar) equates to 13 substitutions, given that 6,682 SNPs was used to estimate it.

For each serovar from each food sample, the number of differences in AMR determinants, plasmid replicons and year of collection were plotted against the number of SNP differences with the closest United Kingdom human isolate (Supplementary figure 19).

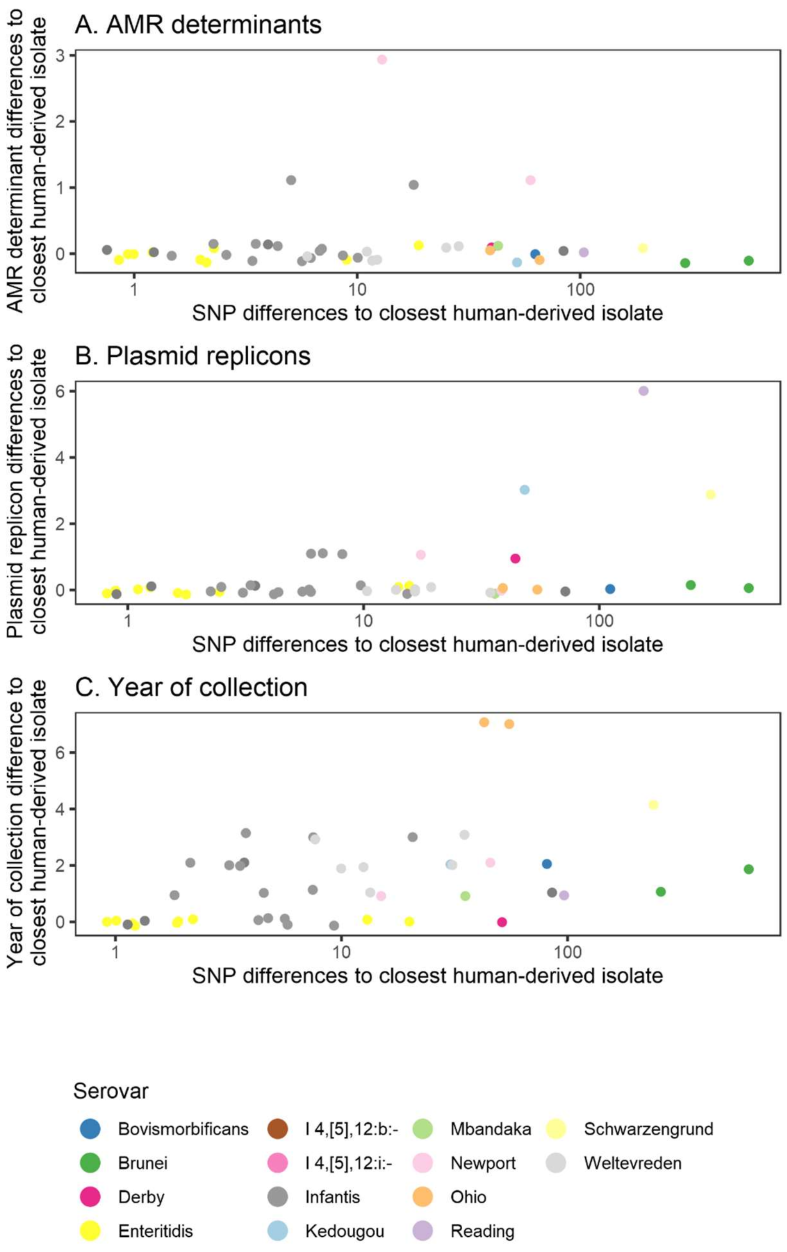

Supplementary figure 19. Jitter plots of the smallest number of AMR determinant (A), plasmid replicon (B), and year of collection (C) differences, and the SNP differences for isolates belonging to serovars from each food surveillance sample and the closest UK human-derived isolate

For chicken samples, 67% (2/3) of butcher samples that tested positive for NTS contained isolates that were less than five SNPs to human UK isolates compared to 63% (17/27) of those from chain supermarkets, but NTS were only cultured from three butcher samples (Supplementary figure 20), and the differences were not significant ($p = 0.90$). For product presentation, 50% (6/12) of chilled chicken samples that tested positive for NTS contained isolates that were within five SNPs to human UK isolates, compared to 72% (13/18) of frozen chicken samples, but the differences were not significant ($p = 0.23$). For country of origin, 44% (4/9) of domestic chicken samples that tested positive for NTS contained isolates that were within five SNPs to human UK isolates, compared to 75% (15/20) of imported chicken samples, but the proportions were not significantly different ($p = 0.12$). However, a larger proportion of imported chicken samples cultured for NTS compared to domestic chicken samples, and a larger proportion of frozen chicken samples cultured for NTS compared to chilled chicken samples. We could not determine the origin of one chicken sample from which NTS was isolated and isolates from this sample were greater than five SNPs to human UK isolates. Taking into consideration NTS culture positive and negative chicken samples, 17% (15/88) of imported chicken samples contained NTS isolates that were within five SNPs to clinical isolates compared to 2.3% (5/214) domestic samples investigated, and these percentages were significantly different ($p < 0.00001$). In addition, 2.7% (6/225) of chilled chicken contained NTS isolates that were within five SNPs to clinical isolates compared to 15% (13/86) of frozen chicken samples, and these percentages were significantly different ($p = 5.3 \times 10^{-5}$). However, 3.1% (2/64) of butcher chicken samples contained NTS isolates that were within five SNPs to clinical isolates compared to 7.1% (17/238) of chain supermarket chicken samples, and these percentages were not significantly different ($p = 0.24$).

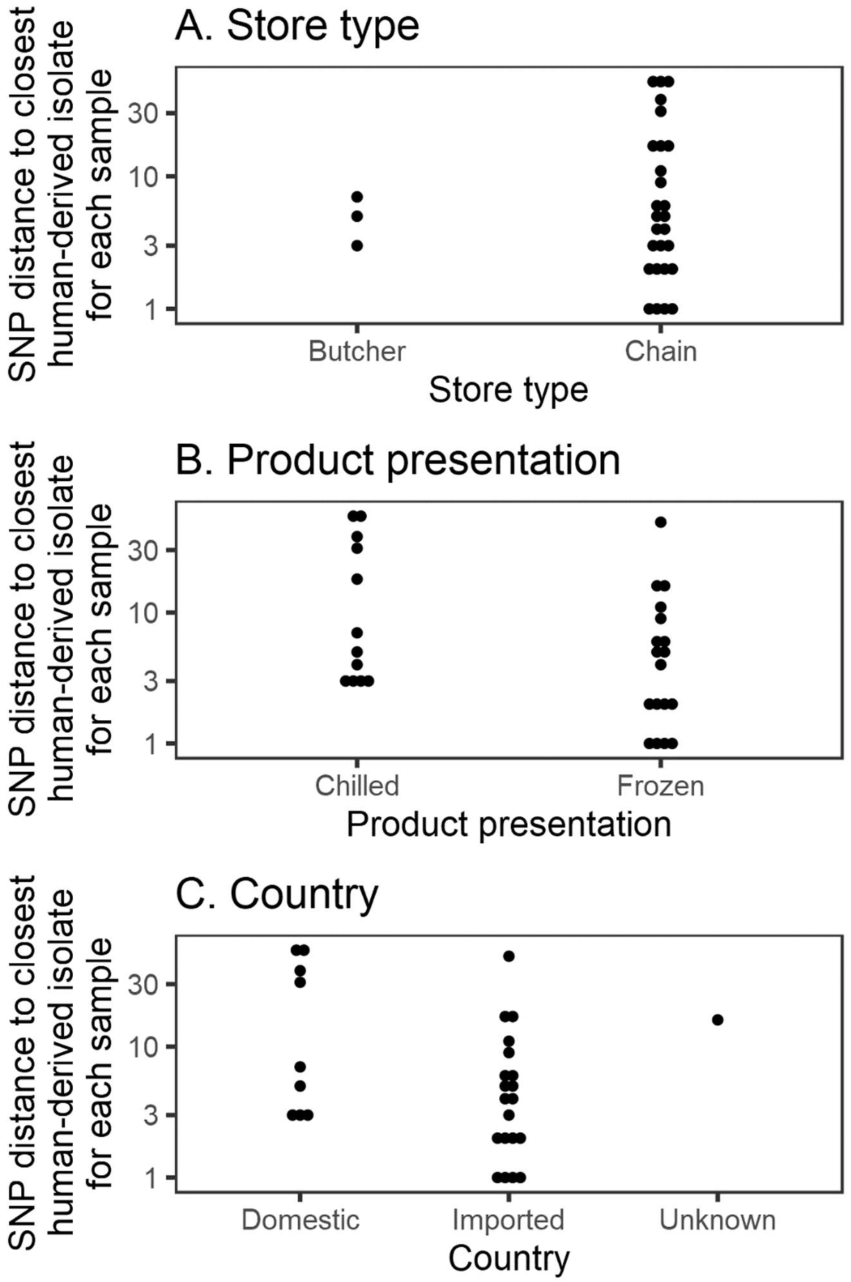

Supplementary figure 20. Dot plots of the number of SNPs between NTS from chicken samples and the closest UK human isolates, separated by store type (A), product presentation (B) and country (C).

Supplementary table 3. AMR determinants identified from each NTS isolate obtained from food in this study.

Isolate	Source	Serovar	AMR determinants
**SA18CH-0043-2**	Chicken	Enteritidis	gyrA (S83Y)
**SA18CH-0043-3**	Chicken	Enteritidis	gyrA (S83Y)
**SA18CH-0043-4**	Chicken	Enteritidis	gyrA (S83Y)

**SA18CH-0043-5**	Chicken	Enteritidis	gyrA (S83Y)
**SA18CH-0043-6**	Chicken	Enteritidis	gyrA (S83Y)
**SA18CH-0043-7**	Chicken	Enteritidis	gyrA (S83Y)
**SA18CH-0043-8**	Chicken	Enteritidis	gyrA (S83Y)
**SA18CH-0053-1**	Chicken	Enteritidis	gyrA (S83Y)
**SA18CH-0053-2**	Chicken	Enteritidis	gyrA (S83Y)
**SA18CH-0053-3**	Chicken	Enteritidis	gyrA (S83Y)
**SA18CH-0053-4**	Chicken	Enteritidis	gyrA (S83Y)
**SA18CH-0053-5**	Chicken	Enteritidis	gyrA (S83Y)
**SA18CH-0053-6**	Chicken	Enteritidis	gyrA (S83Y)
**SA18CH-0053-7**	Chicken	Enteritidis	gyrA (S83Y)
**SA18CH-0060-1**	Chicken	Infantis	aadA1, gyrA (S83Y), qacEdelta1, sul1, tet_A
**SA18CH-0060-2**	Chicken	Infantis	aadA1, gyrA (S83Y), qacEdelta1, sul1, tet_A
**SA18CH-0060-3**	Chicken	Infantis	aadA1, gyrA (S83Y), qacEdelta1, sul1, tet_A
**SA18CH-0060-4**	Chicken	Infantis	aadA1, gyrA (S83Y), qacEdelta1, sul1, tet_A
**SA18CH-0060-5**	Chicken	Infantis	aadA1, gyrA (S83Y), qacEdelta1, sul1, tet_A
**SA18CH-0060-6**	Chicken	Infantis	aadA1, gyrA (S83Y), qacEdelta1, sul1, tet_A
**SA18CH-0062-2**	Chicken	Enteritidis	gyrA (S83Y)
**SA18CH-0062-3**	Chicken	Enteritidis	gyrA (S83Y)
**SA18CH-0062-4**	Chicken	Enteritidis	gyrA (S83Y)
**SA18CH-0062-5**	Chicken	Enteritidis	gyrA (S83Y)
**SA18CH-0062-6**	Chicken	Enteritidis	gyrA (S83Y)
**SA18CH-0062-7**	Chicken	Enteritidis	gyrA (S83Y)
**SA18CH-0062-8**	Chicken	Enteritidis	gyrA (S83Y)
**SA18CH-0070-2**	Chicken	Enteritidis	gyrA (S83Y)
**SA18CH-0070-3**	Chicken	Enteritidis	gyrA (S83Y)
**SA18CH-0070-4**	Chicken	Enteritidis	gyrA (S83Y)
**SA18CH-0070-5**	Chicken	Enteritidis	gyrA (S83Y)
**SA18CH-0070-6**	Chicken	Enteritidis	gyrA (S83Y)
**SA18CH-0073-1**	Chicken	Infantis	aadA1, gyrA (S83Y), qacEdelta1, sul1, tet_A
**SA18CH-0073-2**	Chicken	Infantis	aadA1, gyrA (S83Y), qacEdelta1, sul1, tet_A
**SA18CH-0073-3**	Chicken	Infantis	aadA1, gyrA (S83Y), qacEdelta1, sul1, tet_A
**SA18CH-0073-4**	Chicken	Infantis	aadA1, gyrA (S83Y), qacEdelta1, sul1, tet_A
**SA18CH-0073-5**	Chicken	Infantis	aadA1, gyrA (S83Y), qacEdelta1, sul1, tet_A

**SA18CH-0073-6**	Chicken	Infantis	aadA1, gyrA (S83Y), qacEdelta1, sul1, tet_A
**SA18CH-0087-1**	Chicken	Mbandaka	No AMR determinants
**SA18CH-0087-2**	Chicken	Mbandaka	No AMR determinants
**SA18CH-0087-3**	Chicken	Mbandaka	No AMR determinants
**SA18CH-0087-4**	Chicken	Mbandaka	No AMR determinants
**SA18CH-0087-5**	Chicken	Mbandaka	No AMR determinants
**SA18CH-0087-6**	Chicken	Mbandaka	No AMR determinants
**SA18CH-0087-7**	Chicken	Mbandaka	No AMR determinants
**SA18CH-0087-8**	Chicken	Mbandaka	No AMR determinants
**SA18CH-0089-1**	Chicken	Newport	aadA1, aph_3___Ia, blaTEM_1, gyrA (S83Y), qnrB19, sul2, tet_A
**SA18CH-0089-2**	Chicken	Newport	aadA1, aph_3___Ia, blaTEM_1, gyrA (S83Y), qnrB19, sul2, tet_A
**SA18CH-0089-3**	Chicken	Newport	aadA1, aph_3___Ia, blaTEM_1, gyrA (S83Y), qnrB19, sul2, tet_A
**SA18CH-0089-4**	Chicken	Newport	aadA1, aph_3___Ia, blaTEM_1, gyrA (S83Y), qnrB19, sul2, tet_A
**SA18CH-0089-5**	Chicken	Newport	aadA1, aph_3___Ia, blaTEM_1, gyrA (S83Y), qnrB19, sul2, tet_A
**SA18CH-0105-1**	Chicken	Infantis	aadA1, gyrA (S83Y), qacEdelta1, sul1, tet_A
**SA18CH-0105-2**	Chicken	Infantis	aadA1, gyrA (S83Y), qacEdelta1, sul1, tet_A
**SA18CH-0105-3**	Chicken	Infantis	aadA1, gyrA (S83Y), qacEdelta1, sul1, tet_A
**SA18CH-0105-4**	Chicken	Infantis	aadA1, gyrA (S83Y), qacEdelta1, sul1, tet_A
**SA18CH-0106-1**	Chicken	Infantis	aadA1, aph_3___Ia, dfrA14, gyrA (D87G), qacEdelta1, sul1, tet_A
**SA18CH-0106-2**	Chicken	Infantis	aadA1, aph_3___Ia, dfrA14, gyrA (D87G), qacEdelta1, sul1, tet_A
**SA18CH-0106-3**	Chicken	Infantis	aadA1, aph_3___Ia, dfrA14, gyrA (D87G), qacEdelta1, sul1, tet_A
**SA18CH-0106-4**	Chicken	Infantis	aadA1, aph_3___Ia, dfrA14, gyrA (D87G), qacEdelta1, sul1, tet_A
**SA18CH-0106-5**	Chicken	Infantis	aadA1, aph_3___Ia, dfrA14, gyrA (D87G), qacEdelta1, sul1, tet_A
**SA18CH-0106-6**	Chicken	Infantis	aadA1, aph_3___Ia, dfrA14, gyrA (D87G), qacEdelta1, sul1, tet_A
**SA18CH-0106-7**	Chicken	Infantis	aadA1, aph_3___Ia, dfrA14, gyrA (D87G), qacEdelta1, sul1, tet_A
**SA18CH-0106-8**	Chicken	Infantis	aadA1, aph_3___Ia, dfrA14, gyrA (D87G), qacEdelta1, sul1, tet_A
**SA18CH-0111-1**	Chicken	Infantis	aadA1, gyrA (S83Y), qacEdelta1, sul1, tet_A
**SA18CH-0111-2**	Chicken	Infantis	aadA1, gyrA (S83Y), qacEdelta1, sul1, tet_A

**SA18CH-0111-3**	Chicken	Infantis	aadA1, gyrA (S83Y), qacEdelta1, sul1, tet_A
**SA18CH-0111-4**	Chicken	Infantis	aadA1, gyrA (S83Y), qacEdelta1, sul1, tet_A
**SA18CH-0117-1**	Chicken	Enteritidis	No AMR determinants
**SA18CH-0117-2**	Chicken	Enteritidis	No AMR determinants
**SA18CH-0117-3**	Chicken	Enteritidis	No AMR determinants
**SA18CH-0117-4**	Chicken	Enteritidis	No AMR determinants
**SA18CH-0117-5**	Chicken	Enteritidis	No AMR determinants
**SA18CH-0126-1**	Chicken	Ohio	No AMR determinants
**SA18CH-0126-2**	Chicken	Ohio	No AMR determinants
**SA18CH-0126-3**	Chicken	Ohio	No AMR determinants
**SA18CH-0126-4**	Chicken	Ohio	No AMR determinants
**SA18CH-0126-5**	Chicken	Ohio	No AMR determinants
**SA18CH-0126-6**	Chicken	Ohio	No AMR determinants
**SA18CH-0126-7**	Chicken	Ohio	No AMR determinants
**SA18CH-0126-8**	Chicken	Ohio	No AMR determinants
**SA18CH-0127-1**	Chicken	Infantis	aadA1, gyrA (S83Y), qacEdelta1, sul1, tet_A
**SA18CH-0127-2**	Chicken	Infantis	aadA1, gyrA (S83Y), qacEdelta1, sul1, tet_A
**SA18CH-0127-3**	Chicken	Infantis	aadA1, gyrA (S83Y), qacEdelta1, sul1, tet_A
**SA18CH-0127-4**	Chicken	Infantis	aadA1, gyrA (S83Y), qacEdelta1, sul1, tet_A
**SA18CH-0127-5**	Chicken	Infantis	aadA1, gyrA (S83Y), qacEdelta1, sul1, tet_A
**SA18CH-0127-6**	Chicken	Infantis	aadA1, gyrA (S83Y), qacEdelta1, sul1, tet_A
**SA18CH-0127-7**	Chicken	Infantis	aadA1, gyrA (S83Y), qacEdelta1, sul1, tet_A
**SA18CH-0134-1**	Chicken	Infantis	aadA1, gyrA (S83Y), qacEdelta1, sul1, tet_A
**SA18CH-0134-3**	Chicken	Infantis	aadA1, gyrA (S83Y), qacEdelta1, sul1, tet_A
**SA18CH-0134-5**	Chicken	Infantis	aadA1, gyrA (S83Y), qacEdelta1, sul1, tet_A
**SA18CH-0134-7**	Chicken	Infantis	aadA1, gyrA (S83Y), qacEdelta1, sul1, tet_A
**SA18CH-0138-1**	Chicken	Infantis	aadA1, aph_3___Ia, dfrA14, gyrA (D87G), qacEdelta1, sul1, tet_A
**SA18CH-0138-3**	Chicken	Infantis	aadA1, aph_3___Ia, dfrA14, gyrA (D87G), qacEdelta1, sul1, tet_A
**SA18CH-0138-5**	Chicken	Infantis	aadA1, aph_3___Ia, dfrA14, gyrA (D87G), qacEdelta1, sul1, tet_A
**SA18CH-0138-7**	Chicken	Infantis	aadA1, aph_3___Ia, dfrA14, gyrA (D87G), qacEdelta1, sul1, tet_A

**SA18CH-0146-1**	Chicken	Ohio	No AMR determinants
**SA18CH-0146-3**	Chicken	Ohio	No AMR determinants
**SA18CH-0146-4**	Chicken	Ohio	No AMR determinants
**SA18CH-0146-5**	Chicken	Ohio	No AMR determinants
**SA18CH-0146-7**	Chicken	Ohio	No AMR determinants
**SA18CH-0147-1**	Chicken	Infantis	aadA1, gyrA (S83Y), qacEdelta1, sul1, tet_A
**SA18CH-0147-3**	Chicken	Infantis	aadA1, gyrA (S83Y), qacEdelta1, sul1, tet_A
**SA18CH-0147-5**	Chicken	Infantis	aadA1, gyrA (S83Y), qacEdelta1, sul1, tet_A
**SA18CH-0147-7**	Chicken	Infantis	aadA1, gyrA (S83Y), qacEdelta1, sul1, tet_A
**SA18CH-0150-1**	Chicken	Enteritidis	No AMR determinants
**SA18CH-0150-2**	Chicken	Enteritidis	No AMR determinants
**SA18CH-0150-3**	Chicken	Enteritidis	No AMR determinants
**SA18CH-0150-5**	Chicken	Enteritidis	No AMR determinants
**SA18CH-0163-1**	Chicken	Infantis	aadA1, dfrA14, gyrA (D87G), qacEdelta1, sul1, tet_A
**SA18CH-0163-3**	Chicken	Infantis	aadA1, dfrA14, gyrA (D87G), qacEdelta1, sul1, tet_A
**SA18CH-0163-5**	Chicken	Infantis	aadA1, aph_3___Ia, dfrA14, gyrA (D87G), qacEdelta1, sul1, tet_A
**SA18CH-0163-7**	Chicken	Infantis	aadA1, dfrA14, gyrA (D87G), qacEdelta1, sul1, tet_A
**SA18CH-0180-1**	Chicken	Enteritidis	gyrA (S83Y)
**SA18CH-0240-1**	Chicken	Newport	qnrB19
**SA18CH-0240-3**	Chicken	Newport	qnrB19
**SA18CH-0240-5**	Chicken	Newport	qnrB19
**SA18CH-0240-7**	Chicken	Newport	qnrB19
**SA18CH-0246-1**	Chicken	I 4,[5],12:i:-	aph_3____Ib, aph_6__Id, blaTEM_1, sul2, tet_B
**SA18CH-0246-2**	Chicken	I 4,[5],12:i:-	aph_3____Ib, aph_6__Id, blaTEM_1, sul2, tet_B
**SA18CH-0255-1**	Chicken	Enteritidis	gyrA (S83Y)
**SA18CH-0255-3**	Chicken	Enteritidis	gyrA (S83Y)
**SA18CH-0255-5**	Chicken	Enteritidis	gyrA (S83Y)
**SA18CH-0261-1**	Chicken	Infantis	aadA1, gyrA (D87G), qacEdelta1, sul1, tet_A
**SA18CH-0261-3**	Chicken	Infantis	aadA1, gyrA (D87G), qacEdelta1, sul1, tet_A
**SA18CH-0261-5**	Chicken	Infantis	aadA1, gyrA (D87G), qacEdelta1, sul1, tet_A
**SA18CH-0261-7**	Chicken	Infantis	aadA1, gyrA (D87G), qacEdelta1, sul1, tet_A
**SA18CH-0265-1**	Chicken	Kedougou	No AMR determinants

**SA18CH-0265-3**	Chicken	Kedougou	No AMR determinants
**SA18CH-0265-5**	Chicken	Kedougou	No AMR determinants
**SA18CH-0273-1**	Chicken	Infantis	aadA1, aph_3___Ia, dfrA14, gyrA (D87G), qacEdelta1, sul1, tet_A
**SA18CH-0273-3**	Chicken	Infantis	aadA1, aph_3___Ia, dfrA14, gyrA (D87G), qacEdelta1, sul1, tet_A
**SA18CH-0273-5**	Chicken	Infantis	aadA1, aph_3___Ia, dfrA14, gyrA (D87G), qacEdelta1, sul1, tet_A
**SA18CH-0273-7**	Chicken	Infantis	aadA1, aph_3___Ia, dfrA14, gyrA (D87G), qacEdelta1, sul1, tet_A
**SA18CH-0278-1**	Chicken	Infantis	aadA1, floR, gyrA (S83Y), qacEdelta1, sul1, tet_A
**SA18CH-0278-2**	Chicken	Infantis	aadA1, floR, gyrA (S83Y), qacEdelta1, sul1, tet_A
**SA18CH-0278-3**	Chicken	Infantis	gyrA (S83Y)
**SA18CH-0278-4**	Chicken	Infantis	gyrA (S83Y)
**SA18CH-0304-1**	Chicken	Infantis	aadA1, blaTEM_1, gyrA (S83Y), qacEdelta1, qnrS1, sul1, tet_A
**SA18CH-0304-3**	Chicken	Infantis	aadA1, blaTEM_1, gyrA (S83Y), qacEdelta1, qnrS1, sul1, tet_A
**SA18CH-0304-5**	Chicken	Infantis	aadA1, blaTEM_1, gyrA (S83Y), qacEdelta1, qnrS1, sul1, tet_A
**SA18CH-0311-1**	Chicken	Enteritidis	gyrA (S83Y)
**SA18CH-0311-3**	Chicken	Enteritidis	gyrA (S83Y)
**SA18CH-0311-5**	Chicken	Enteritidis	gyrA (S83Y)
**SA18PK-0042-1**	Pork	I 4,[5],12:i:-	aph_3____Ib, aph_6__Id, blaTEM_1, sul2, tet_B
**SA18PK-0042-2**	Pork	I 4,[5],12:i:-	aph_3____Ib, aph_6__Id, blaTEM_1, sul2, tet_B
**SA18PK-0042-3**	Pork	I 4,[5],12:i:-	aph_3____Ib, aph_6__Id, blaTEM_1, sul2, tet_B
**SA18PK-0137-1**	Pork	I 4,[5],12:i:-	aph_3____Ib, aph_6__Id, blaTEM_1, sul2, tet_B
**SA18PK-0137-3**	Pork	I 4,[5],12:i:-	aph_3____Ib, aph_6__Id, blaTEM_1, sul2, tet_B
**SA18PK-0137-5**	Pork	I 4,[5],12:i:-	aph_3____Ib, aph_6__Id, blaTEM_1, sul2, tet_B
**SA18PK-0137-7**	Pork	I 4,[5],12:i:-	aph_3____Ib, aph_6__Id, blaTEM_1, sul2, tet_B
**SA18PK-0242-1**	Pork	Reading	fosA7
**SA18PK-0242-3**	Pork	Reading	fosA7
**SA18PK-0242-5**	Pork	Reading	fosA7
**SA18PK-0242-7**	Pork	Reading	fosA7
**SA18PK-0299-1**	Pork	Derby	fosA7
**SA18PK-0299-2**	Pork	Derby	fosA7
**SA18PK-0299-3**	Pork	Derby	fosA7
**SA18PK-0299-4**	Pork	Derby	fosA7

**SA18PK-0299-5**	Pork	Derby	fosA7
**SA18PK-0299-6**	Pork	Derby	fosA7
**SA18PK-0299-7**	Pork	Derby	fosA7
**SA18PK-0299-8**	Pork	Derby	fosA7
**SA18PR-0007-1**	Prawns	Weltevreden	No AMR determinants
**SA18PR-0007-2**	Prawns	Weltevreden	No AMR determinants
**SA18PR-0007-3**	Prawns	Weltevreden	No AMR determinants
**SA18PR-0007-4**	Prawns	Weltevreden	No AMR determinants
**SA18PR-0007-5**	Prawns	Bovismorbificans	No AMR determinants
**SA18PR-0007-6**	Prawns	Bovismorbificans	No AMR determinants
**SA18PR-0007-7**	Prawns	Bovismorbificans	No AMR determinants
**SA18PR-0007-8**	Prawns	Bovismorbificans	No AMR determinants
**SA18PR-0037-1**	Prawns	Weltevreden	No AMR determinants
**SA18PR-0037-2**	Prawns	Weltevreden	No AMR determinants
**SA18PR-0037-3**	Prawns	Weltevreden	No AMR determinants
**SA18PR-0037-4**	Prawns	Weltevreden	No AMR determinants
**SA18PR-0037-5**	Prawns	Weltevreden	No AMR determinants
**SA18PR-0037-6**	Prawns	Weltevreden	No AMR determinants
**SA18PR-0067-2**	Prawns	Weltevreden	No AMR determinants
**SA18PR-0067-3**	Prawns	Weltevreden	No AMR determinants
**SA18PR-0067-4**	Prawns	Weltevreden	No AMR determinants
**SA18PR-0067-5**	Prawns	Weltevreden	No AMR determinants
**SA18PR-0067-6**	Prawns	Weltevreden	No AMR determinants
**SA18PR-0126-3**	Prawns	Brunei	No AMR determinants
**SA18PR-0126-4**	Prawns	Brunei	No AMR determinants
**SA18PR-0126-5**	Prawns	Brunei	No AMR determinants
**SA18PR-0126-6**	Prawns	Brunei	No AMR determinants
**SA18PR-0151-1**	Prawns	I 4,[5],12:b:-	No AMR determinants
**SA18PR-0151-2**	Prawns	Brunei	No AMR determinants
**SA18PR-0151-3**	Prawns	Weltevreden	No AMR determinants
**SA18PR-0151-4**	Prawns	I 4,[5],12:b:-	No AMR determinants
**SA18PR-0151-5**	Prawns	I 4,[5],12:b:-	No AMR determinants
**SA18PR-0151-6**	Prawns	Weltevreden	No AMR determinants
**SA18PR-0191-1**	Prawns	Weltevreden	No AMR determinants
**SA18PR-0191-3**	Prawns	Weltevreden	No AMR determinants
**SA18PR-0198-1**	Prawns	Weltevreden	No AMR determinants
**SA18PR-0198-2**	Prawns	Weltevreden	No AMR determinants
**SA18PR-0198-3**	Prawns	Weltevreden	No AMR determinants
**SA18PR-0238-1**	Prawns	Schwarzengrund	No AMR determinants
**SA18PR-0238-3**	Prawns	Schwarzengrund	No AMR determinants
**SA18PR-0238-7**	Prawns	Schwarzengrund	No AMR determinants
